# Supplementary material for: Affect-congruent attention modulates generalized reward expectations
Source: PLoS Comput Biol. 2023 Dec 21;19(12):e1011707. doi: 10.1371/journal.pcbi.1011707 (PMC10781156; doi:10.1371/journal.pcbi.1011707)
Supplement: S1 Text — All supporting information can be found within the associated PDF file. (PDF) [file pcbi.1011707.s001.pdf]

Supporting information, ‘Affect-congruent attention modulates generalized reward expectations’

Daniel Bennett[* 1], Angela Radulescu[* 2], Sam Zorowitz[3], Valkyrie Felso[4], Yael Niv[3,5]

[*]Equal contribution
[1]School of Psychological Sciences, Monash University, Australia
[2]Department of Psychiatry, Icahn School of Medicine at Mount Sinai, New York, NY, USA
[3]Princeton Neuroscience Institute, Princeton University, USA
[4]Max Planck Institute for Intelligent Systems, Tübingen, Germany
[5]Department of Psychology, Princeton University, USA

Section A: Validation of affect-induction procedure

This study manipulated participants' affect using video clips with happy, neutral, or sad content. In some cases, these videos were drawn from extant affect-induction libraries. In others, however, videos were chosen especially for this study (see Table S1). We therefore conducted an online validation experiment to verify that our chosen videos produced the desired change in participants' self-reported affect.

Validation method

448 participants (50 per video; data from 2 participants unavailable due to server error) were recruited via Amazon Mechanical Turk (mean age (SD) = 37.33 (11.57); 181 females, 265 males, and 2 participants who did not report a binary gender). Each participant's mood was probed twice using an Affective Slider: once before watching the video, and once after. For both probes, participants reported both valence and arousal. The effect of videos on participants' mood was measured as the difference of pre- and post-video mood ratings.

To ensure that we only measured affective responses from participants who actively engaged with the videos, participants completed two comprehension checks immediately after the video: one based on identifying a still image from the video, and one based on comprehension of the events of the video. Data from 56 participants (12.5% of sample) who failed either of these comprehension checks were excluded from further analysis.

All participants provided informed consent, and this study was approved by the Princeton University IRB (#4452). Total time taken per participant was approximately 5 minutes, and each participant was paid USD $1 for their time. The webpage used custom JavaScript code built using the JSPsych library.

Validation results

In the pre-video mood probe, participants reported mildly positive levels of both valence (mean = 0.64 on a 0 - 1 scale where neutral was coded as 0.5; SD = 0.21) and arousal (mean = 0.63, SD = 0.23). Figure S1 presents video-related changes from this baseline level.

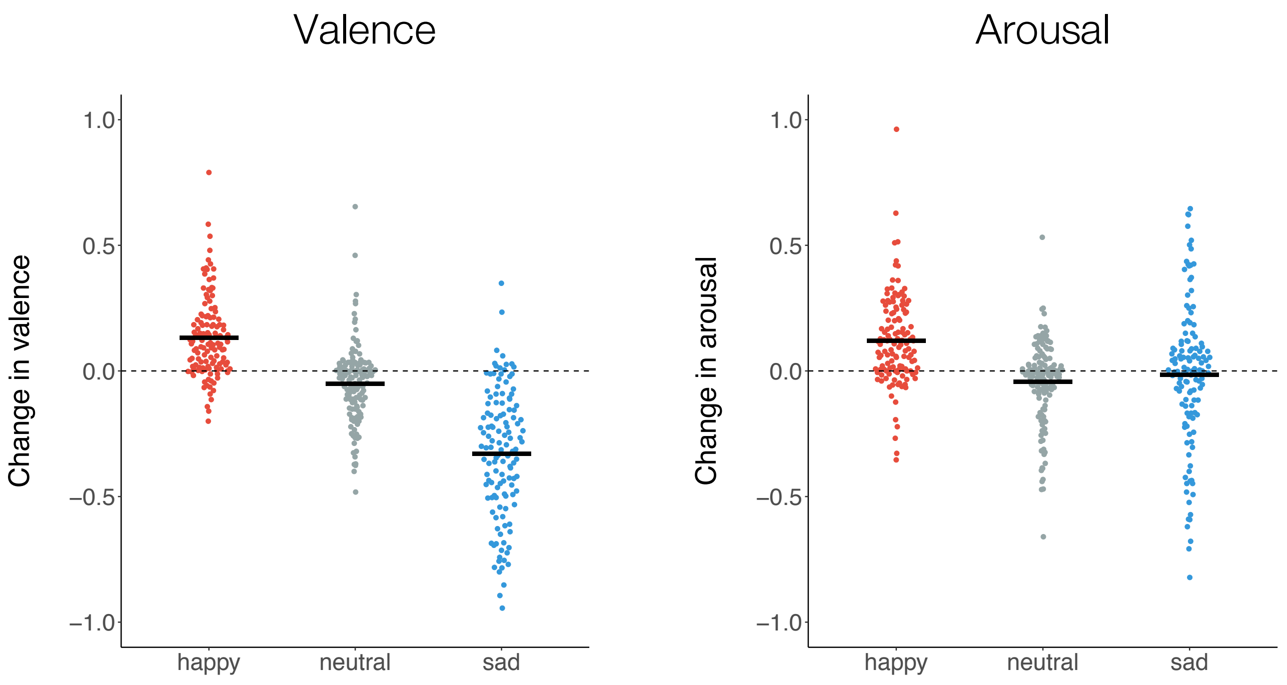

Figure S1: **Video-related changes in affect (post- minus pre-video rating).** Left: changes in self-reported valence of mood. Left: changes in self-reported arousal. Red: happy videos; grey: neutral videos; blue: sad videos. Each dot represents change in mood for one participant. Horizontal black lines denote the mean of each condition. Ratings are normalised by the length of the rating scale (i.e., +1/-1 are largest possible positive/negative changes in mood).

As expected, the valence of mood was significantly modulated by the videos (happy versus neutral: β = 0.18, $p < .001$; sad versus neutral: β = -0.28, $p < .001$). The larger effect size for sad videos than happy videos is in line with a recent meta-analysis suggesting a 'negativity bias' in affect induction procedures [1]. In addition, there was was a small but significant tendency for neutral videos to produce a negative shift in mood (β = -0.05, $p < .01$). Inspection of raw data suggested that this was due to a tendency for participants to report exactly neutral mood (i.e., exactly 0.5 on the slider) after neutral videos. Since baseline mood levels were moderately positive, this tendency manifested as a modest negative shift for neutral videos. Happy videos significantly increased arousal relative to neutral videos (β = 0.16, $p < .001$), and there there was no difference between the effect of sad videos and the effect of neutral videos on arousal (β = 0.03, $p = .30$).

Table S1 presents characteristics of individual videos, including quantification of video-specific effects on the valence of mood. Within each video condition, the effects of the different videos were reasonably similar. In particular, the variance between different videos in each condition was markedly smaller than the variance between participants or between conditions.

Table S1: Videos used in affect induction procedure

Condition	Video #	Length	Description	Source	Mean valence [SD]
	1	90 s	A figure skater celebrating	2002 Olympic broadcast	0.12 [0.14]
Happy	2	90 s	A girl getting a kitten for her birthday	Publicly available online	0.18 [0.17]
	3	91 s	Two dancers in dinosaur costumes	Publicly available online	0.11 [0.16]
	4	89 s	Two men talking in a courtroom	*All the President's Men*	-0.01 [0.15]
Neutral	5	90 s	Two women talking in a clothing store	*Hannah and Her Sisters*	-0.05 [0.16]
	6	83 s	A car driving in a city; a man at a desk	*The Lover* & *Three Colours: Blue*	-0.09 [0.15]
	7	90 s	A woman dying after a car crash	*City of Angels*	-0.29 [0.29]
Sad	8	90 s	A boy crying at his father's funeral	*The Champ*	-0.37 [0.24]
	9	90 s	A girl crying at her friend's funeral	*My Girl*	-0.32 [0.24]

Video 1 sourced from Gruber et al. (2008) [2].

Videos 4 and 5 sourced from Hewig et al. (2005) [3].

Videos 6–9 sourced from Schaefer et al. (2010) [4].

Video 6 was created by concatenating two shorter videos.

Valence calculated as post-video valence rating minus pre-video valence rating.

Section B: Choice pairs in compound generalization

Table S2 below presents a detailed overview of the 96 trials that participants completed across the three blocks of the compound generalization phase.

Note that for each stimulus pair type (e.g., M vs. L/H), there were multiple ways of constructing a choice pair (since there were two low-value cues, two medium-value cues, and two high-value cues). The task was designed in this way so as to prevent excessive repetition of visually identical choice pairs. Table S1 therefore details the number of visually distinct configurations corresponding to each stimulus pair ('N cue configurations') as well as the the number of times that each configuration was repeated within a pair type.

Table S2: Choice pairs for choice generalization phase of the task

Stimulus pair type	N cue configurations	Repetitions per configuration	N trials
M vs. L/H	8	2	16
M/M vs. L/H	4	6	24
M vs. L/M	4	2	8
M vs. M/H	4	2	8
L/M vs. L/H	8	2	16
L/M vs. M/H	8	1	8
L/H vs. M/H	8	2	16

L: Low-value cue (25% reward probability) M: Medium-value cue (50% reward probability) H: High-value cue (75% reward probability)

Section C: Supplemental analyses of behavioural data

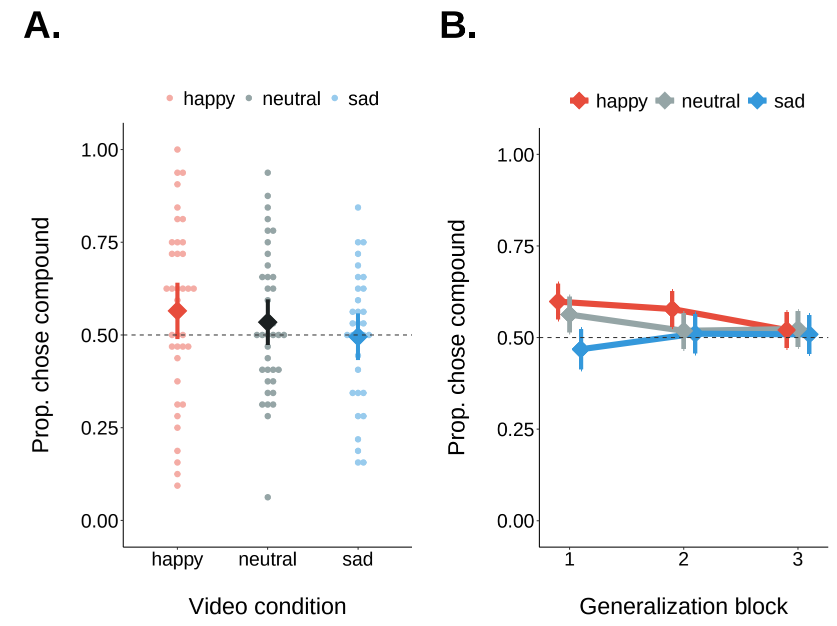

Figure S2: Overall preference for compound (vs. simple) stimuli across participants (**A**) and compound generalization blocks (**B**). Diamond markers denote group means and their 95% confidence intervals.

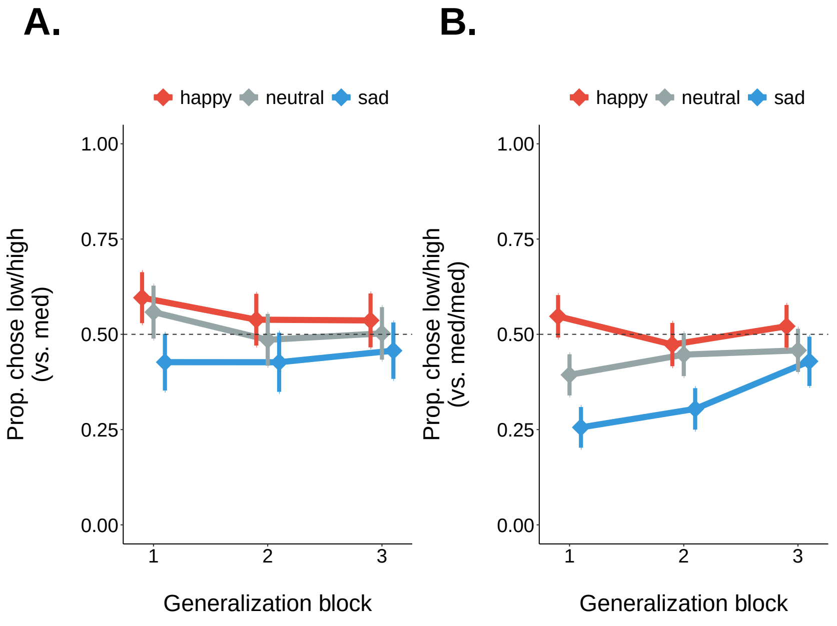

Figure S3: Empirical proportion of choices of the L/H compound as a function of affect condition and block number in simple probe trials (**A**; choice between L/H compound stimulus and M simple stimulus) and compound probe trials (**B**; choice between L/H compound stimulus and M/M compound stimulus). Diamond markers denote the mean of each condition and its 95 % confidence interval; background points indicate choice proportions for individual participants

Finally, we conducted separate analyses of both choice and reaction time data for the M vs. L/M and M vs. M/H trials. We did not find any effect of affect-induction group

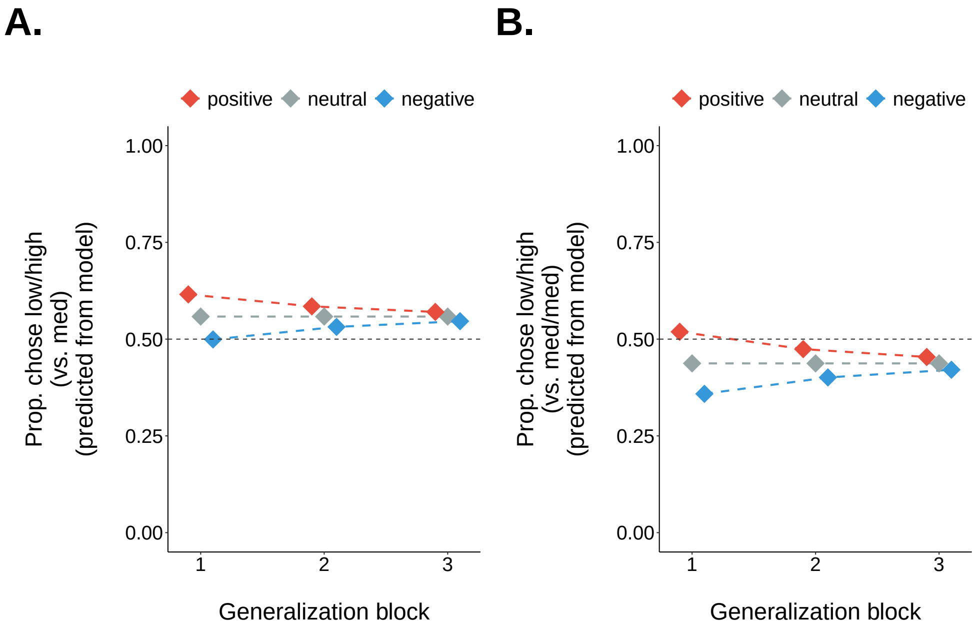

Figure S4: Posterior predictive check for behavioural model: predicted proportion of choices of the L/H compound as a function of affect condition and block number in simple probe trials (**A**; choice between L/H compound stimulus and M simple stimulus) and compound probe trials (**B**; choice between L/H compound stimulus and M/M compound stimulus). Compare with Figure S9 above.

on choice data either for the M vs. L/M trials ($\beta = 0.39, p = .10$, mixed-effects logistic regression) or for the M vs. M/H trials ($\beta = -0.01, p = .96$, mixed-effects logistic regression). Similarly, we did not find any effect of affect-induction group on reaction time either for the M vs. L/M trials ($\beta = 0.06, p = .37$, mixed-effects linear regression) or for the M vs. M/H trials ($\beta = -0.01, p = .86$, mixed-effects linear regression). We note that the experiment was not powered for these analyses, and so it is somewhat difficult to interpret this pattern of effects because of the small per-participant sample size for each of these trial types (each participant completed only eight M vs. L/M trials and eight M vs. M/H trials across the entire experiment, compared to sixteen M vs. L/H and twenty-four M/M vs. L/H trials). Because the primary model-agnostic dependent variable for our analyses was behaviour on the 'probe trials' (i.e., M vs. L/H and M/Mvs. L/H), we did not optimise our task for detecting effects in these other trials (though our modelling results predict that we should observe effects of affect-induction group on choice given sufficient statistical power).

Section D: Model and parameter recovery analyses

We conducted a model recovery analysis to determine whether the models that we compared were in principle identifiable on the basis of the behavioural data that we collected. The results of this model recovery analysis are presented in Figure S5, and are consistent with acceptable levels of model identification across the models that we considered.

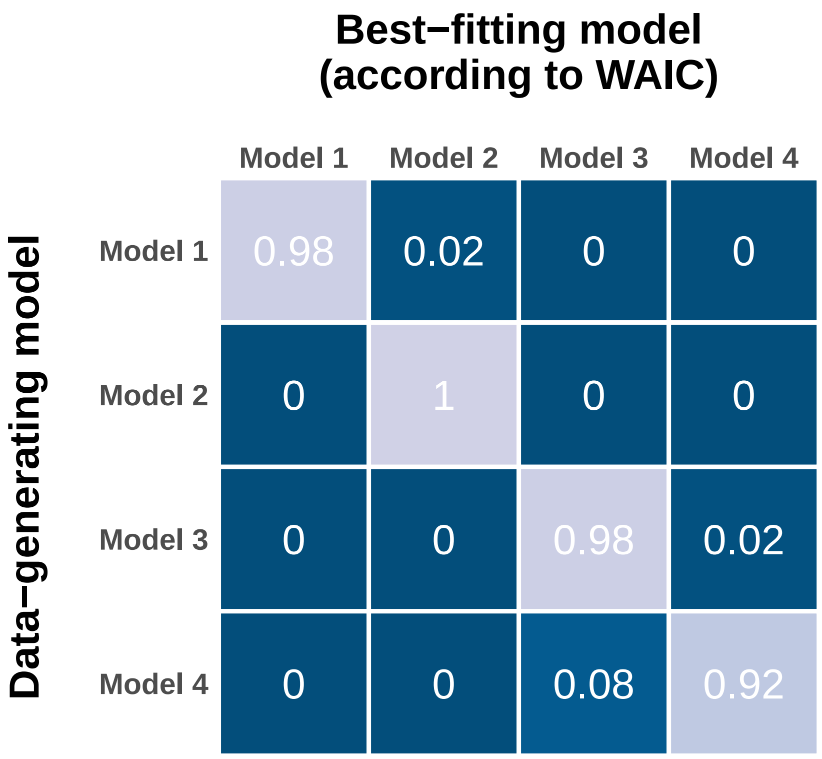

Figure S5: Model recovery results for each of the four models presented in Table 1. 50 datasets were generated from each of the four models (rows of matrix) and the best-fitting model for each dataset was selected using the WAIC statistic (columns). The numbers in each cell of the matrix depict the proportion of generated datasets that were identified as having been generated by a given model. Under perfect model recovery, all numbers on the matrix diagonal would be equal to 1.

We also conducted a parameter recovery analysis. Here, we simulated a behavioral dataset of 100 synthetic 'participants' for each of the models presented in Table 1. To ensure that synthetic data were similar to the observed data, parameter values for synthetic participants were drawn from the estimated group-level posterior distributions from the models as fit to actual data. We then used the estimation procedure described in the main text to estimate all free parameters for the synthetic data generated from each model.

Parameter recovery results are presented in Table S3, and graphically in Figure S6 for the best-fitting Model 4. There are two noteworthy features of these results: first, parameters were recovered at acceptable ($> .50$) levels for all parameters, albeit with less accuracy for ϕ and α_V than for the softmax inverse temperature parameter β. Second, for each parameter, estimation accuracy was similar across all models in which the parameter was fit (columns in Table S3), suggesting that the accuracy of estimation of each parameter was not substantially influenced by the simultaneous estimation of the other parameters in the model.

Table S3: Summary of parameter recovery results for each of the models in Table 1.

Model	β	ϕ	α_V
1	.92	-	-
2	.86	.61	-
3	.93	-	.65
4	.94	.59	.64

Parameter recovery quantified as the Spearman rank-order correlation between generated and estimated parameter values.

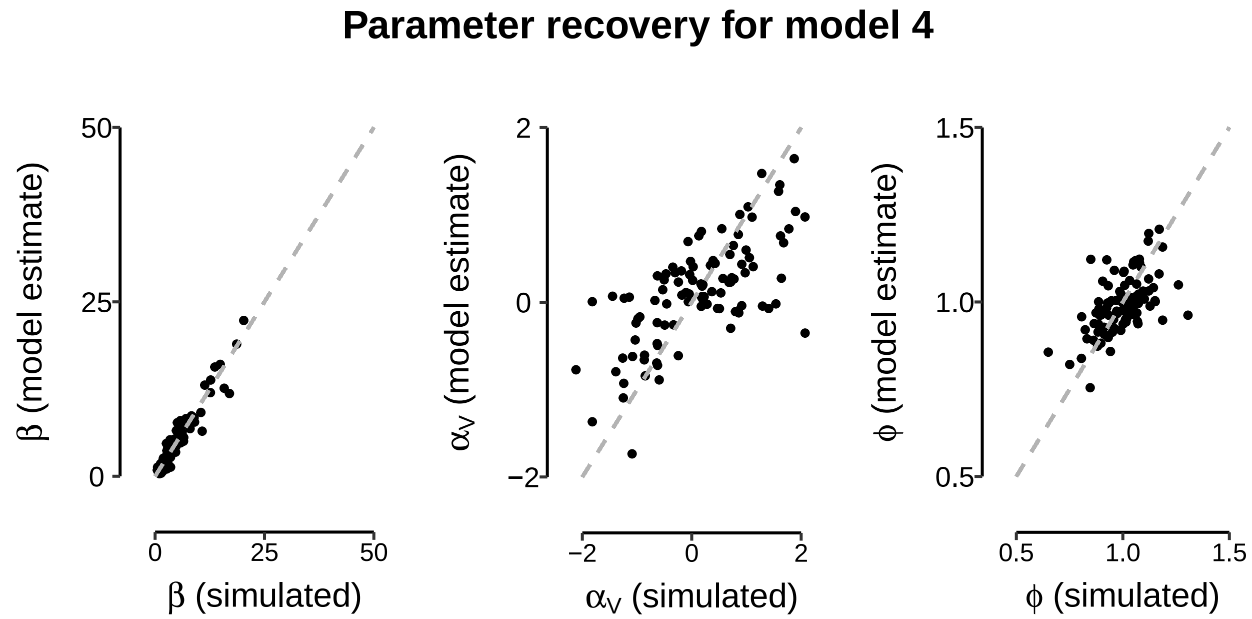

Figure S6: Parameter recovery results for each free parameter in Model 4. Under perfect parameter recovery, all points would fall on the grey x=y line.

Section E: Supplementary model analyses

In appraising the effects of affect on the model parameters α_V and ϕ, we modelled the effect of affect using a continuous coding scheme (i.e., sad affect coded as -1, neutral affect coded as 0, happy affect coded as 1). This coding convention implicitly assumes that the effects of happy and sad affect inductions are equal in magnitude but opposite in sign. This is a parsimonious way of investigating the effects of affect on parameters, but it is not the only coding scheme that we might have chosen. For instance, rather than being equal in magnitude, it is also possible that the sad affect induction may have had a stronger effect on behaviour than the happy induction, or vice versa (the former would be consistent with the generalised tendency for negative affect inductions to be stronger than positive affect inductions; see, e.g., [1]).

To ensure that we were justified in modelling the effect of affect using a continuous coding scheme, we conducted an additional round of computational model comparison (Table S4). In these comparisons, the baseline model implementing a continuous coding scheme (denoted 4c-λ) was compared with 15 competing models in which the effect of affect on each parameter was either coded using distinct offset parameters for happy and sad affect inductions (denoted by '+ and −' in Table S4), with an offset parameter for the happy induction only (denoted by '+', and corresponding to the hypothesis that only that happy affect induction influenced a particular parameter), or with an offset parameter for the sad induction only (denoted by '−', and corresponding to the hypothesis that only that sad affect induction influenced a particular parameter). As in previous computational modelling analyses, we compared models using the WAIC statistic. In cases where two models had a statistically equivalent WAIC value (i.e., a difference in WAIC less than two standard errors of the WAIC difference between the two models), we broke ties by selecting the model with fewer parameters per participant.

The results of this analysis indicated that several models produced a statistically equivalent fit to the data. When ties were broken according to model parsimony, we found that none of the alternative models provided a better account of the effects of the affect induction on behaviour than the original model 4c-λ. This result validates the assumptions entailed by our continuous coding scheme for the effects of affect on α_V and ϕ.

Table S4: Summary of supplementary model comparison to test the assumption (instantiated in Model 4c-λ) of equal and opposite effects of affect across happy and sad affect inductions.

Model	Modulation of α_V	Modulation of ϕ	n free parameters per participant	WAIC	ΔWAIC (Std. Err.)
4c-λ	continuous	continuous	6	13508.3	5.9 (3.1)
4c-λ-v2	continuous	+ and −	7	13507.7	5.3 (3.8)
4c-λ-v3	continuous	+	6	13541.7	39.3 (14.2)
4c-λ-v4	continuous	−	6	13564.0	61.6 (16.3)
4c-λ-v5	+ and −	continuous	7	13502.4	0.4 (2.5)
4c-λ-v6	+ and −	+ and −	8	13502.9	0 (-)
4c-λ-v7	+ and −	+	7	13538.2	35.7 (13.4)
4c-λ-v8	+ and −	−	7	13564.8	62.4 (16.4)
4c-λ-v9	+	continuous	6	13532.8	30.4 (8.8)
4c-λ-v10	+	+ and −	7	13532.5	29.1 (8.9)
4c-λ-v11	+	+	6	13573.2	70.8 (16.6)
4c-λ-v12	+	−	6	13588.4	86.0 (18.3)
4c-λ-v13	−	continuous	6	13523.1	20.7 (10.7)
4c-λ-v14	−	+ and −	7	13525.7	23.3 (10.8)
4c-λ-v15	−	+	6	13555.5	53.0 (17.3)
4c-λ-v16	−	−	6	13629.5	127.1 (22.4)

WAIC: Watanabe-Akaike Information Criterion, presented on a deviance scale such that lower numbers indicate better model fit. ΔWAIC: the difference between the WAIC of each model and that of the best-fitting model.

Section F: Supplementary eye-tracking analyses

In the eye-tracking analyses reported in the main text, we operationalized attention to low- and high-value cues by measuring the proportion of time spent fixating on these stimuli *as a proportion of all the cues present on the entire screen.* An alternative way of operationalizing this construct would be to compute proportion of looking time to each cue as a proportion of looking time to all the cues present *within the stimulus of which it was a part.*

When we instead operationalized overt visual attention in this way, there was no significant interaction between experiment phase (pre- vs. post-induction) and affect-congruency, $\chi^2(1) = 0.02, p = .90$.

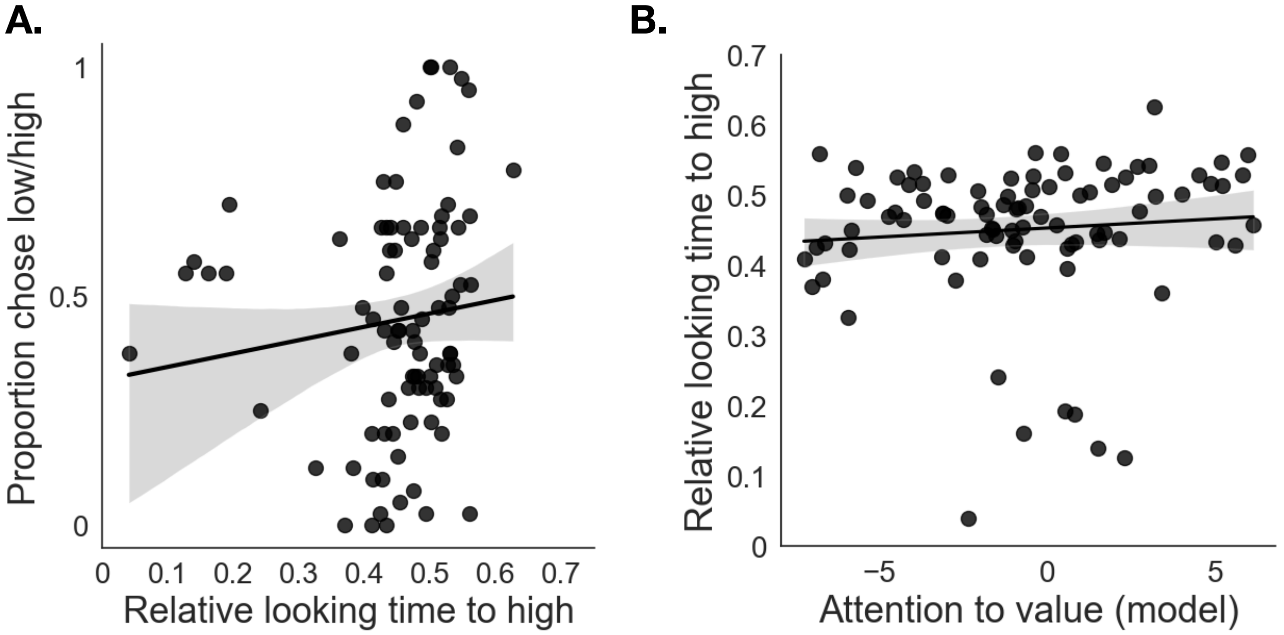

Figure S7: Results from analyses in Figure 5C and 5D in the main text, repeated with normalization done within the *L/H* stimulus. **A:** Relative looking time to the *H* stimulus significantly predicted the rendency to choose the *L/H* stimulus ($\chi^2(1) = 7.54, p = 0.006$). **B:** Attention to value estimated from the model was not a significant predictor of relative looking time to the *H* stimulus ($\chi^2(1) = 0.70, p = 0.40$).

Section G: Eye-tracking data quality assurance

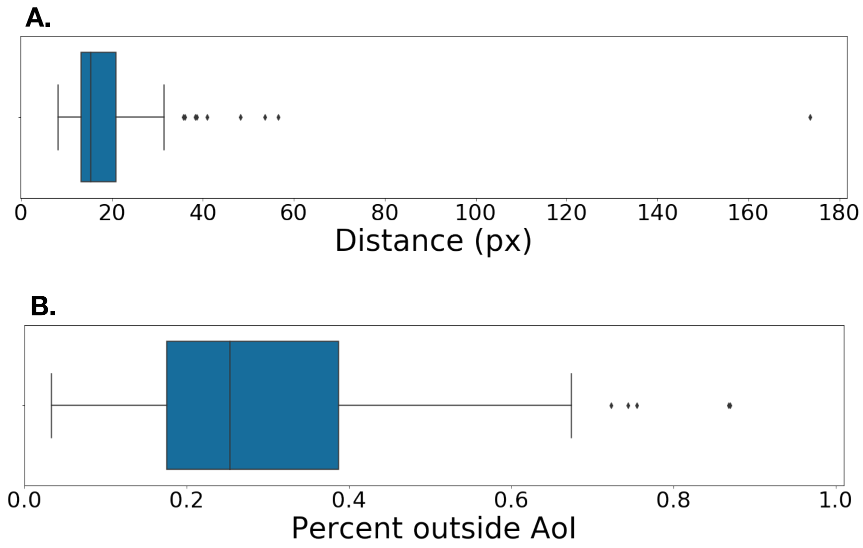

Figure S8: **Eye-tracking quality assurance. A.** Criterion 1: distance between the position of custom AoI centers hand-picked per participant and block by two independent raters. **B.** Criterion 2: percentage of samples that fall outside a valid AoI. In addition to participants excluded due to failure to learn the task, participants who were more than 1.5 $\times$ IQR above 3rd quartile in either criterion were excluded from the analysis.

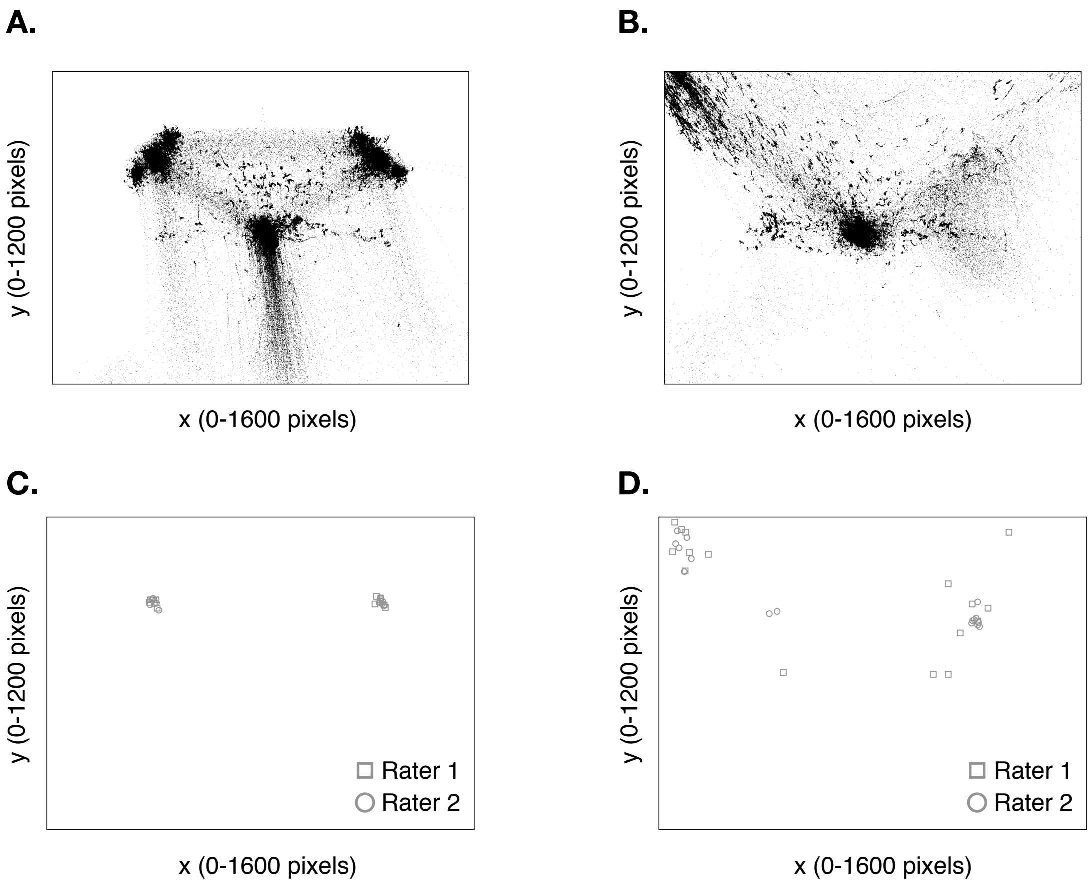

Figure S9: **Comparison between datasets with the highest and lowest rater agreement. A.** Raw data for the participant with the highest rater agreement. **B.** Raw data for the participant with the lowest rater agreement. **C.** Position of custom AoI centers hand-picked per block by two independent raters for the participant shown in (A). **D.** Position of custom AoI centers hand-picked per block by two independent raters for the participant shown in (B).

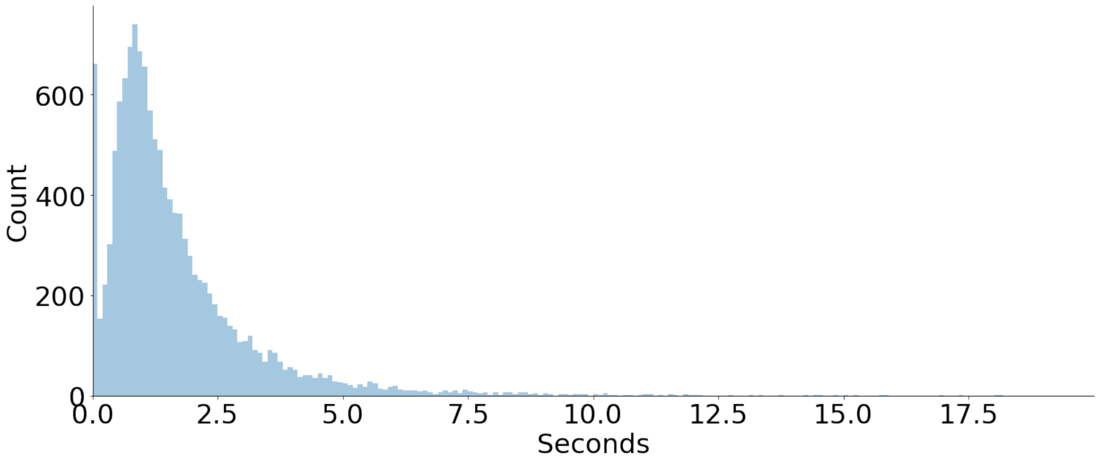

Figure S10: **Total looking time to valid cues before choice.** Histogram of total looking time spent looking at valid cues before choosing, computed across all trials and all participants. Data during this time interval was used to compute relative looking times to each simple cue.

Section H: Custom Area of Interest (AoI) centering

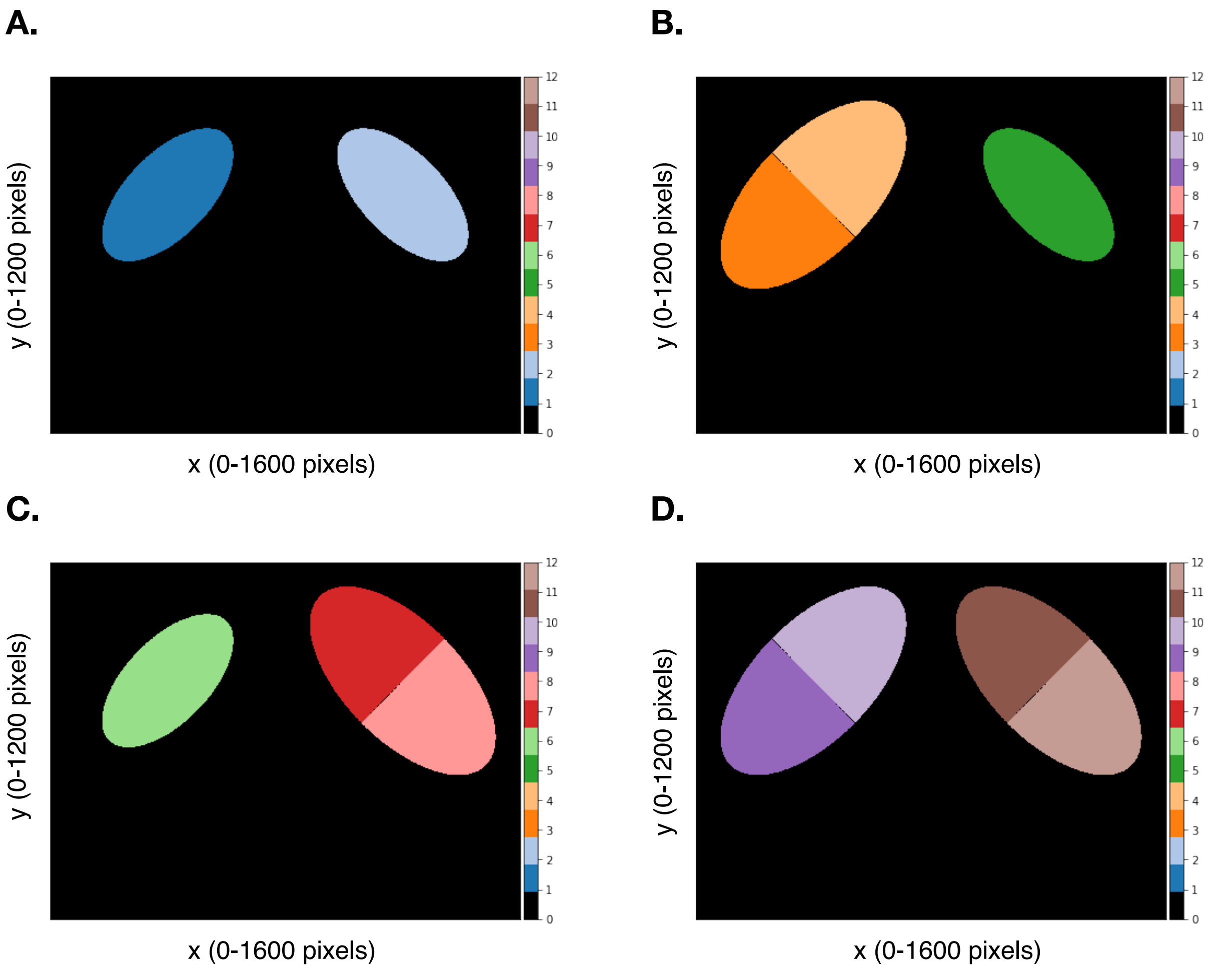

Figure S11: **Possible AoI configurations.** Elliptical AOI configurations for simple vs. simple (**A**), compound vs. simple (**B**), simple vs. compound (**C**) and compound vs. compound (**D**) trials.

To mitigate drift between participants and across blocks, two independent raters hand-picked the centers of the left and right AoIs, separately for each participant and block.

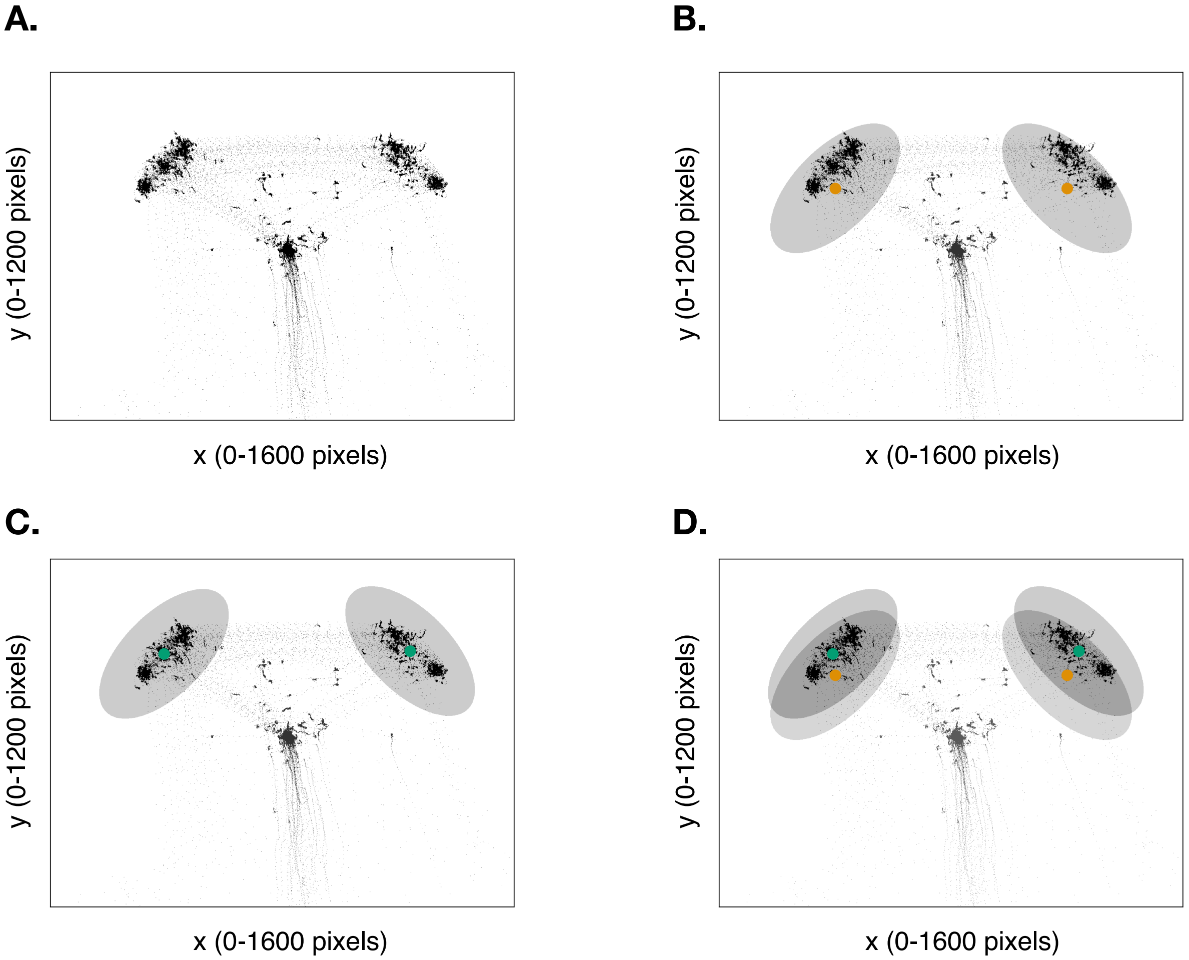

Figure S12: **Custom AoI centering.** **A.** Sample eye-tracking data from a participant making choices during one block of the test phase. **B.** Data and elliptical AoIs with centers coinciding to those of the left and right planet stimuli (base centers). **C.** Data and elliptical AoIs with hand-picked centers (custom centers). **D.** Comparison of base (orange) and custom (green) centers.

Section H: Supplemental analyses of mood data

Table S5: Spearman correlation matrix for baseline self-reported valence and arousal ($N = 109$)

	Baseline valence	Baseline arousal	Hypomania (GBI)	Depression (GBI)	Positive affect (PANAS)
Baseline arousal	.25 **	-	-	-	-
Hypomania (GBI)	-.07	.10	-	-	-
Depression (GBI)	-.27 **	.12	.58 ***	-	-
Positive affect (PANAS)	.29 **	.31 **	.25 *	.01	-
Negative affect (PANAS)	.04	.22 *	.27 **	.13	.77 ***

***: $p < .001$; **: $p < .01$; *: $p < .05$

Table S6: Spearman correlation matrix for size of sad affect induction on self-reported valence and arousal ($N = 32$)

	Hypomania (GBI)	Depression (GBI)	Positive affect (PANAS)	Negative affect (PANAS)	Valence change
Valence change	.02	.22	.08	.16	-
Arousal change	.13	-.07	.38 *	.30	.05

*: $p < .05$

Section I: Between-groups demographic comparison

Table S7 below presents demographic statistics as a function of affect induction condition.

Table S7: Demographic data

	Full sample	Happy	Neutral	Sad
N participants	120	40	40	40
Age	21.31	22.31	20.30	21.37
Gender	77 F, 43 M	26 F, 14 M	29 F, 11 M	22 F, 18 M
GBI Hypomania	31.77	33.65	31.33	31.15
GBI Depression	74.02	77.80	74.93	71.60
PANAS positive affect	21.18	22.03	19.82	21.78
PANAS negative affect	17.24	17.52	16.76	17.47

As expected, there were no between-groups differences in age (one-way ANOVA: $F(2, 102) = 2.10, p = .13$), gender (chi-square test of independence: $\chi^2(2) = 2.34, p = .31$), hypomania (Kruskal-Wallis test: $\chi^2(2) = 1.37, p = .50$), depression (Kruskal-Wallis test: $\chi^2(2) = 0.65, p = .72$), positive affect (one-way ANOVA: $F(2, 105) = 2.70, p = .07$), or negative affect (one-way ANOVA: $F(2, 105) = 0.32, p = .65$).

References

1. Joseph, D. L., Chan, M. Y., Heintzelman, S. J., Tay, L., Diener, E., & Scotney, V. S. (2020). The manipulation of affect: A meta-analysis of affect induction procedures. *Psychological Bulletin, 146*(4), 355-375.

2. Gruber, J., Johnson, S. L., Oveis, C., & Keltner, D. (2008). Risk for mania and positive emotional responding: Too much of a good thing? *Emotion, 8*(1), 23–33

3. Hewig, J., Hagemann, D., Seifert, J., Gollwitzer, M., Naumann, E., & Bartussek, D. (2005). A revised film set for the induction of basic emotions. *Cognition and Emotion, 19*(7), 1095–1109.

4. Schaefer, A., Nils, F., Sanchez, X., & Philippot, P. (2010). Assessing the effectiveness of a large database of emotion-eliciting films: A new tool for emotion researchers. *Cognition and Emotion, 24*(7), 1153–1172.
