# Supplementary material for: Affect-congruent attention modulates generalized reward expectations
Source: PLoS Comput Biol. 2023 Dec 21;19(12):e1011707. doi: 10.1371/journal.pcbi.1011707 (PMC10781156; doi:10.1371/journal.pcbi.1011707)
Supplement: Attachment — Submitted filename: response.pdf [file pcbi.1011707.s002.pdf]

Response to Reviews

Formatting note: in the following document, comments from reviewers are printed in **bold typeface.** Excerpts from the revised manuscript are presented in italics, with additions being presented in underlined typeface and deletions being presented in ~~strikethrough~~ typeface. We have renumbered reviewers' comments in places to aid clarity of responding.

Reviewer #1

This article investigates the effects of affect inductions on reward-based choice via an attentional mechanism. The authors induce negative or positive affect using short videos, then examine behavior on a generalization task with ambiguously valued stimuli. They find that positive affect leads to more optimistic valuation of the ambiguous compound stimuli, and that this change is accompanied by a shift in looking time to the high value stimuli, at least for one of the conditions. The authors also employ a simple model to estimate attention from the choice data. Here they find that attention is modulated by affect.

Overall I found the article to be quite interesting and well written. I appreciated the mix of affective and cognitive psychology, along with eye-tracking and computational modeling. It is unfortunate that there isn't better agreement between the modeling and the eye-tracking analysis (though see my comments below), but I don't think that this should be held against the authors. The experiments were well designed and the hypotheses made perfect sense. It just turns out that the story may be more complex than expected. If the authors can address the few questions/concerns that I have, I would be happy to endorse publication.

Thank you for these encouraging words. We appreciate your judging the work by its design and research questions, letting the data determine the results as they do.

R1.1 The authors focus their analysis on choices between L/H compound stimuli and either M or M/M stimuli. This is obviously where they expect to see the biggest effect of their affect manipulation on choice. However, shouldn't we also expect to see differences in the M vs. L/M and M vs. M/H trials as well, either in choices or in reaction times? Positive affect participants should be slower in M vs. L/M and faster in M vs. M/H compared to negative affect participants. Similarly, positive affect participants should be faster than negative affect participants in L/M vs. L/H and negative affect participants should be faster than positive affect participants in L/H vs. M/H. There should be no effect of affect on L/M vs. M/H.

Thank you for this excellent suggestion. We have now conducted both of the analyses suggested by the reviewer. Unfortunately, for both choice data and for reaction times, we did not observe the pattern of results predicted by the reviewer:

- We did not find any effect of affect-induction group on choice data either for the M vs. L/M trials (β = 0.39, *p* = .10, mixed-effects logistic regression) or for the M vs. M/H trials (β = -0.01, *p* = .96, mixed-effects logistic regression).
- Similarly, we did not find any effect of affect-induction group on reaction time either for the M vs. L/M trials (β = 0.06, *p* = .37, mixed-effects linear regression) or for the M vs. M/H trials (β = -0.01, *p* = .86, mixed-effects linear regression).

We note that the experiment was not powered for these analyses, and so it is somewhat difficult to interpret this pattern of effects because of the small per-participant sample size for each of these trial types (each participant completed only eight M vs. L/M trials and eight M vs. M/H trials across the entire experiment, compared to sixteen M vs. L/Hand twenty-four M/M vs. L/H trials). Because the primary model-agnostic dependent variable for our analyses was behaviour on the 'probe trials' (M vs. L/H and M/M vs. L/H), we did not optimise our task for detecting effects in the trials highlighted by the reviewer (though we agree that our modelling results predict that we should observe the choice effects predicted by the reviewer given sufficient statistical power). Consequently, it is difficult to know whether the non-significant results in the trial subtype analyses requested by the reviewer is because of a lack of statistical power versus a true null effect in these trials. As such, we have not included these results in the current manuscript, but are happy to add them to the supplementary material if you think this would be helpful to readers.

R1.2 It also wasn't clear which analyses were run on the probe trials vs. the non-probe trials. Were the eye-tracking analyses run on all trials or just the probe trials? If it is the former, perhaps this explains why there is a discrepancy between those results and the modeling results, which seem to have been run on all the data.

We thank the reviewer for this comment. The eye-tracking analyses that were run in service of testing the main hypothesis (Fig. 5C, 5D, and 6) were run on probe trials only. We have added the following clarification text on Page 15 to make this point clearer:

> <u>*To provide mechanistic insight into how affect modulates feature-based attention and its effect on choice, we next turned to analyses of looking time in compound-generalization* ***probe trials*** *(that is, trials in which participants chose between the L/H compound and either a M or M/M stimulus).*</u>

R1.3 Regarding the modeling - I had a couple of related concerns.

R1.3.1 The finding that delta_phi was largely negative has me concerned about a tradeoff between that parameter and the alpha parameter. They appear to be pulling in opposite directions, suggesting that the overall change might be negligible.

We thank the reviewer for raising this point. In addressing this comment, we first note that although numerically negative, the $\Delta\phi$ parameter was not credibly different from 0 at a group level (posterior median = -0.026, Bayesian HDI = [-0.07, 0.01]). Nevertheless, given that our modelling results depend on the interpretation of the α_V parameter, we agree that it is important to rule out a potential trade-off between the effects of the affect induction on the ϕ parameter and the effects of the affect induction on the α_V parameter.

If the parameters ϕ and α_V are indeed trading off against each other when fit simultaneously in the same model, we would expect estimates of each parameter to be substantially different if they were fit in a model that did not include the other parameter (i.e., we would predict differences in $\Delta\phi$ when it is estimated in a model with vs. a model without a $\Delta\alpha_V$ parameter, and differences in $\Delta\alpha_V$ when it is estimated in a model with vs. a model without a $\Delta\phi$ parameter). The existing suite of models that we compared allowed us to directly test for this. Consistent with dissociable effects of $\Delta\alpha_V$ and $\Delta\phi$ on behavior, we found that each parameter had a very similar estimated posterior distribution regardless of whether or not the other parameter was estimated alongside it. Specifically, when the ϕ parameter was fit in the joint model we estimated a group-level mean ϕ value of 1.14 (90% Bayesian Highest Density Interval (HDI) [1.11, 1.19]) and an affect-related change ($\Delta\phi$) of -0.027 (HDI [-0.07, 0.01]), compared with a group-level mean of 1.15 (HDI [1.11, 1.20]) and an affect-related change of -0.026 (HDI [-0.08, 0.03]) when it was fit by itself (in Model *4a-λ*; see Table 3). Similarly, when α_V parameter was fit in the joint model we estimated an overall group-level mean α_V of -0.52 (HDI [-1.25, 0.21]) and an affect-related change ($\Delta\alpha_V$) of 0.68 (HDI [0.03, 1.42]), compared with a group-level median of -0.53 (HDI [-1.23, 0.19]) and an affect-related change of 0.63 (HDI [-0.06, 1.34]) when it was fit by itself in Model *4b-λ*.

Taken together, these results give us confidence that $\Delta\alpha_V$ and $\Delta\phi$ did not substantially trade off against one another when they were fit simultaneously. We thank the reviewer for prompting us to examine this aspect of our data more closely (see also our response to Reviewer 2's comment R2.7).

R1.3.2 This made me wonder why the authors chose the specific functional form for the attention weights in equation 2. Why not instead simply estimate weights A_H, A_M, and A_L in each condition? This would eliminate equation 2 altogether and any assumptions about the functional form of attention. With only three value levels this should barely increase model complexity.

We thank the reviewer for this comment, and provide more rationale for our chosen approach below.

We could indeed have estimated attention weights separately for each stimulus value level to remain agnostic with respect to the functional form of attention. We chose an alternative approach (using a functional form) for practical and interpretation considerations. First, had we modelled behaviour with separate attention weights A_H , A_M , and A_L , as suggested by the reviewer, we would have had to estimate three additional parameters ΔA_H , ΔA_M , and ΔA_L to determine the effects of the affect induction on behaviour. This would have been highly challenging because the attention weights, A_H , A_M , and A_L would need to sum to one for each participant (in order for the compound preference parameter ϕ to remain identifiable). Consequently, an increase in any one of the ΔA parameters requires a decrease in the other two ΔA parameters. This would have led to issues of parameter identifiability and interpretability, because model estimates of each ΔA parameter would not be independent of estimated values for the other ΔA parameters. These issues do not arise in our chosen approach precisely because of the constraints we imposed regarding linearity of attention as a function of value. Therefore, although we agree that the reviewer's suggested approach would have been a reasonable approach to modelling the data in the absence of an affect induction, our research question necessitated the interpretation of changes in model parameters as a function of participants' affect-induction condition. We submit that our chosen modelling approach was chosen appropriately for answering this research question.

Separately, we note that we conducted an additional set of model comparison analyses in which we sought to test our models' assumptions about the functional form of attention. As described in Supplementary Section S10 and on page 14 of the revised manuscript, the results of these analyses supported our assumption that the effects of positive and negative affect on attention were symmetric and in opposite directions. These results give us further confidence in our models' assumptions about the functional form of attention.

R1.4 Regarding the eye-tracking - I didn't understand the need for manual analysis here. Do the results substantially change without this drift correction? Or without this weighting by total fixation time? The EyeLink 1000 Plus should not require such

corrections, but perhaps it was necessary in this case. If so, more explanation would be helpful.

Thank you for asking for this additional clarification. We took special care with drift because we were looking to measure attention *within* a compound stimulus, where even small drift can easily lead to mislabeling component simple cues. Moreover, drift can vary significantly between participants and over time (compare Fig. S5A and S5B). As an example, for the participant whose data are shown in Fig. S5B, vertical drift is much larger than the distance between compound cues, thus would lead to mislabeling if not corrected. Our manual rater-agreement metric captured this data quality discrepancy (compare Fig. S5C and S5D), and allowed us to formulate a quantitative criterion for excluding participants who have excessive drift (Fig. S4A). For participants for whom drift was moderate, the custom correction allowed us to correct for drift while preserving the relative distribution of fixations across AoIs (Fig. S3D). Since this decision was made prior to running the hypothesis-driven analyses, we have opted not to re-run the eye-tracking analysis without this pre-processing step, to not introduce more comparisons and therefore inflate the possibility of type II errors.

Given your concern may be shared by others who are familiar with the EyeLink 1000, we have now added additional clarification of this step in the Methods section, on page 27:

> *We chose this manual quality-assurance method because drift can vary significantly between participants and over time (Supplementary Figure S5A and B), and can lead to mislabeling of within-compound attention if drift is substantial. Our manual rater-agreement metric captured this data-quality discrepancy (compare Supplementary Figure S5C and S5D) and allowed us to formulate a quantitative criterion for excluding participants who have excessive drift (Supplementary Figure S4A). For participants for whom drift was moderate, we used custom correction to correct for drift while preserving the relative distribution of fixations across AoIs.*

The weighting by total fixation time in the eye-tracking mixed-effects analysis (Fig. 6) is a separate analytical choice that we made in order to account for how we computed the main measure of attention (relative looking time). Our reasoning was that shorter trials will include fewer samples for estimating latent proportions of time spent looking at each cue. As such, we expect the estimates for the latent proportions will be noisier in trials with lower total fixation time. The weighting in the mixed-effects model is a statistical choice that accounts for this additional source of noise in our measure of attention.

Minor comments:

R1.5 I thought that the opening example with the diner was ill chosen, in particular the notion that a high price would lead to a lower reward expectation. We know that high prices actually lead people to expect, and experience, higher rewards - see Hilke Plassmann's fMRI research on wine tasting.

This is a good point, and we agree that a different example would better illustrate our point (see also our response to Reviewer 2's comment R2.1). In the revised manuscript, we have replaced the restaurant example with an example involving less ambiguous stimulus features, namely deciding whether to rent an apartment.

Updated text in Introduction:

> *~~In choosing whether to visit a new restaurant, for example, a diner might consider features such as type of cuisine, the chef's reputation, and the price of meals.~~ In choosing whether to rent an apartment, for example, a prospective renter may consider features such as its floorspace, the quality of its furnishings, and the neighbourhood. Using a weighted combination of these features, they can form a generalized reward expectation ~~for the restaurant~~ (i.e., a reward expectation formed via feature-based generalization) ~~even before they eat there (Shulz et al., 2019).~~ that determines their willingness to rent the apartment.*
>
> *How people form generalized reward expectations for a novel stimulus depends on how they allocate attention to its different features. For example, if our ~~diner~~ prospective renter were to attend more to positively valenced stimulus features (i.e., features of the apartment that are predictive of reward, such as ~~a preferred style of cuisine~~ good-quality furnishings and appliances), they would form a higher reward expectation for the ~~restaurant~~ apartment than if they attended more to its negatively valenced features (such as a ~~high price~~ dangerous neighbourhood or small floorspace).*

R1.6 Ideally Figure 2F would be done for the three conditions separately, perhaps using colored dots here as well?

This is an excellent suggestion. Points in Figure 2F are now coloured by condition (using the same color legend as in subplot A-E).

R1.7 Table 2 - please indicate how standard errors were computed for delta-WAIC. The table also makes reference to a “Model 8”. I think the authors mean Model 4?

We apologise for these omissions, and thank you for noticing the model-numbering typo. Table 2 has now been revised to add the requested information. Specifically, we state that standard errors were computed for ∆WAIC using the approximation method developed by Vehtari, Gelman & Gabry (2017) and implemented in the *loo* package for R.

Updated Table Note for Table 2:

> *∆WAIC: the difference between the WAIC of each model and that of the best-fitting model (model ~~8~~4), calculated as per Vehtari, Gelman, & Gabry, 2017 using the loo package for R.*

R1.8 On page 11 it would be helpful to include the equation for the decay lambda, otherwise one has to flip over to the Methods section to be able to interpret it.

We thank the reviewer for this suggestion. As requested, this section has been revised to include the equation for decay.

Updated text on page 12:

> *In addition, in the best-fitting model, the strength of affect’s influence on these parameters decayed over successive compound-generalization blocks~~ (decaying according to the parameter λ)~~. Specifically, we assumed that this effect decayed according to a power-law decay function with parameter λ. For instance, for $\Delta\alpha_V$ the affect-related change in α_V, the effective parameter value in a given block was computed as $\alpha_V^{effective} = \alpha_V + \Delta\alpha_V \times \lambda^{block} \times$ Affect (where Affect is a dummy-coded categorical variable denoting the participant’s affect-induction condition with neutral affect as the reference condition; see Equation 7 in Methods for further information), such that the effect of affect decayed across blocks.*

R1.9 The authors might find it helpful to reference work on attribute-based attention: Fisher 2021 in OBHDP; Yang and Krajbich 2022 in Psychological Review.

We thank the reviewer for this recommendation. The suggested citations have now been included in the paragraph of the discussion in which we contextualise results with respect to other recent work on computational models of attention and choice.

Updated text in Discussion, page 22:

> *Finally, our results point to the influence of mood-congruent attentional biases when forming generalized reward expectations. How might such an attentional mechanism be implemented? Attention and memory-based processes both play a role in generalization (Radulescu et al., 2021). These accounts are not incompatible: because generalization by definition requires switching between (internal) attention to memory and (external) attention to the sensory environment, one way mood-congruent attentional biases could arise is by directing attention to memories as a function of their valence (Poskanzer and Aly, 2023; Gresch et al., 2023). This suggests it might be beneficial to integrate affective biases into sampling-based models of value-based and attribute-based attention. This suggests it might be beneficial to integrate affective biases into sampling-based models of value-based and attribute-based attention (Anderson, Laurent, & Yantis, 2012; Fisher, 2021; Yang & Krajbich, 2023). Sampling-based models have suggested that attentional effects during value computation, and people's overt fixation patterns, reflect evidence accumulation over internal beliefs about the probability of reward (Krajbich et al., 2010; Krajbich & Rangel, 2011; Krajbich et al., 2012; Callaway & Griffiths, 2019).*

We would like to further acknowledge that while at the time our data was written up for publication, a multi-attribute version of sequential sampling models was not readily available, recent advances in that domain that the reviewer points out potentially make it possible to now answer some of these follow-up questions. While this is a direction we are very interested in pursuing, we opted to publish the initial hypothesis-driven results separately, and leave further exploratory analyses on the same dataset to future re-analysis.

<u>**Reviewer #2**</u>

This is a great paper — very clearly written, well-motivated, and beneficial both to basic and applied work on modeling affect, its relation to decision making, and what the two might reveal about latent mechanisms of psychopathology. I hope my comments below help in making final refinements to this paper and the larger project of understanding mechanisms of affect.

R2.1 "For example, if our diner were to attend more to positively valenced stimulus features (i.e., features that are predictive of reward, such as a preferred style of cuisine), they would form a higher reward expectation for the restaurant than if they attended more to its negatively valenced features (such as a high price)"

Although the example makes complete sense, it assumes that value in inherent to stimulus features, and not, for example, conditional on goals. Consider a situation where for instance someone was inferring the quality of the restaurant, and high prices were indicative of such quality. Price then becomes positively valenced. In line with succeeding comments in the Discussion on the ecological validity of the current paradigm compared to other popular emotion/RL experiments (because rightfully it takes into account that complex states have multiple features to which one could selectively attend, and are integrated in determining state value), I think it's useful to take into account that the reward function may shift as goals shift.

We thank the reviewer for their comment, which echoes a comment made by Reviewer 1 about the potential correlation between price and quality in restaurants. As further detailed in our response to comment R1.5 above, we have replaced the restaurant example with the example of renting an apartment that has both good and bad features.

R2.2 Is there variation in comparing high and low mean options? For example, is there evidence that a 75/25 option is treated as worse than 75/50? We would expect this pattern even if people simply attended to the highest-value option — so it would be good to see that when highest-value option is held constant between options, the mean value still matters.

We agree that this is an important point to verify. We had indeed predicted that participants would show a preference for the 50/75 compound stimulus over the 25/75 compound stimulus, and a preference for the 25/75 stimulus over the 25/50 stimulus:

"A qualitative prediction of such a linear rule is that the value of a compound stimulus should be proportional to the mean of the values of its constituent cues (e.g. an L/H [25/75] stimulus should be treated as more valuable than an L/M [25/50] stimulus, but less valuable than an M/H [50/75] stimulus" (Page 7)

However, although we found this to be the case on average (Figure 2C), we did not explicitly test whether it was the case in the specific trial types we mentioned, and which the reviewer identifies here. In the revised manuscript we now explicitly report these contrasts, showing that we do indeed observe the predicted pattern of preferences in these choice trials.

New analyses reported in Results, page 7:

In particular, we observed the predicted qualitative pattern for the choices described above: participants showed a significant preference for the higher-mean L/H stimulus over the lower-mean L/M stimulus when these were the two choice options presented (L/H stimulus chosen on 63% of such trials; $p < .001$, one-sample t-test against chance), and a significant preference for the M/H stimulus over the L/H stimulus (M/H stimulus chosen on 70.2% of trials; $p < .001$, one-sample t-test against chance).

R2.3 "We conducted a model-recovery analysis to ensure that our experimental design was able to differentiate between these models"

Which models? "These" implies referents that were already stated, but I don't seem them until after this sentence.

We thank the reviewer for identifying this inconsistency. We have rewritten this sentence to explain its referents more clearly.

Updated text, page 10:

We conducted a model-recovery analysis to ensure that our experimental design was able to differentiate between ~~these models~~ models in which different combinations of these parameters were free to vary (see Table 1).

R2.4 You mention individual difference analyses that were non-significant in the main text. It would be good to know roughly what you probed for and why you did so in the main text, as it may not only be of general interest, but meaningfully relate to

statements you make below regarding psychopathology and affect-induced attentional biases.

We agree, and have provided the requested information in the Results and Methods sections of the revised manuscript.

Updated text in Results, pages 3-4:

> *We also collected several self-report measures quantifying individual differences in current mood state and potential symptoms of mood disorders (trait depression and hypomania as well as state positive and negative affect; see Methods for further information), since we reasoned that these individual differences might moderate the effects of an affect induction on behavior (S. L. Johnson, 2005; Gruber et al., 2021).*

Updated text in Methods, page 23:

> *Participants completed a behavioral task with concurrent eye-tracking recording, as well as self-report measures of trait depression and hypomania (the General Behavior Inventory (GBI); Depue et al., 1981) and current positive and negative affect (the Positive and Negative Affect Schedule (PANAS); Watson, Clark, & Tellegen, 1988). These individual-difference measures were included because they were identified as potential moderators of the effects of the affect induction on behavior.*

R2.5 As a general clarification, it would be good to know how the statement — "We focused our analysis on two kinds of choice trials…" — involving compound and simple trial types speaks to the data that went into all analyses. Does that mean that computational analyses also only were on these subset of trials?

We thank the reviewer for this comment, which is similar to R1's comment #2. For convenience, we reproduce the response here: the eye-tracking analyses that were run in service of testing the main hypothesis (Fig. 5C, 5D, and 6) were run on probe trials only. We have added the following clarification text on Page 15 to make this point clearer:

> *We next turned to analyses of looking time in compound-generalization **probe trials** (that is, trials in which participants chose between the L/H compound and either a M or M/M stimulus).*

In contrast, computational analyses of behavior were done on all compound generalization trials. We consider this an advantage to our approach, because while the impact of attention on choice can only be directly measured in probe trials for which participants should be

indifferent between the options, computational modeling allows us to use all the data to estimate attentional biases across all choice types.

R2.6 Is the preference for compound stimuli suggestive of the possibility that attention tends to be biased towards the higher-valued option in the compound stimuli? Is there a tradeoff between this parameter and the attention weights? Or does this mean people preferentially choose lower-valued compound stimuli even when it isn't possible for the value to be higher than the simple stimulus regardless of biased attention? For example, do you have evidence to show that the preference for compound stimuli occurs at a greater rate beyond chance that individuals choose a 25/50 stimulus over a single 75 stimulus? Or even 25/50 over a 50 stimulus? I'm not even sure if the modeling incorporated these trials, but they may be useful in comparing your winning model to an alternative that simply decides preferentially based off of either the maximum or minimum value in the compound's pair of features. I don't believe this latter model was tested (this could be a third form of feature integration besides averaging or summing over them).

We agree that the additional choice types described by the reviewer (25/50 vs. 75, 25/50 vs. 50) may provide interesting additional insights regarding the cognitive processes underlying compound generalisation. Unfortunately, however, in the interests of optimising our task design for investigating probe trials, we only tested simple/compound cue pairs in which the simple cue had a value of 50% (see Supplementary Table S1). Nevertheless, in these trials we did indeed find that participants:

A. Chose the 50% simple stimulus significantly <u>more</u> frequently in 50 vs. 25/50 trials (mean choice proportion = 61.2%) than in 50 vs. 25/75 trials (mean choice proportion = 48.3%; $t(116) = 5.13$, $p < .001$, paired samples *t*-test), and
B. Chose the 50% simple stimulus significantly <u>less</u> frequently in 50 vs. 50/75 trials (mean choice proportion = 24.9%) than in 50 vs. 25/75 trials (mean choice proportion = 48.3%; $t(116) = -9.30$, $p < .001$, paired samples *t*-test)

These results provide a quantitative indication that participants did indeed base their decisions on both cues within a compound stimulus, rather than simply considering the cue with the maximum or minimum value. Given these results, therefore, we did not conduct a separate modelling analysis looking at only max/min cue attention weighting (since any such model would not be able to explain the observed results).

We thank the reviewer for suggesting these additional analyses, which are now reported in the Results section of the revised manuscript.

Added text in Results, page 7:

> *For simple-versus-compound trials, we found that participants' choice behavior was sensitive to the value of both cues within each compound: we found that the M simple stimulus was chosen significantly more frequently in M vs. L/M trials (mean choice proportion = 61.2%) than in M vs. L/H trials (mean choice proportion = 48.3%; t(116) = 5.13, p < .001, paired samples t-test). By contrast, participants chose the M simple stimulus significantly less frequently in M vs. M/H trials (mean choice proportion = 24.9%) than in M vs. L/H trials 25/75 trials (t(116) = −9.30, p < .001, paired samples t-test). These results indicate that participants did not simply attend to the highest- or lowest-value cue in a compound, but considered both constituent cues when estimating the value of a compound stimulus.*

Regarding the potential trade-off between the attention-weighting parameter α_V and the compound preference parameter ϕ, we provide further information in response to comment R2.7 below, as well as in response to Reviewer 1's comment R1.3.1 above.

R2.7 This point relates to a question I had about the robustness of the modeling. Did you, in addition to model comparison, endeavor to recover the parameters of your model? This would certainly help answer whether in fact there is tradeoff in the preference for compound over simple stimuli and the attentional weighting parameters.

We thank the reviewer for raising this important point (which echoes comment R1.3.1 by Reviewer 1). As part of the process of validating our computational models we did indeed conduct a parameter recovery exercise. In the revised manuscript we have included the results of these analyses in Section S9, alongside the results of the model recovery exercise.

Briefly, the results of this analysis indicated, first, that parameters were recovered at acceptable levels for all three parameters of the best-fitting model. Second, this analysis also indicated that for the ϕ and α_V parameters (respectively quantifying compound preference and attention weighting) the overall accuracy of parameter recovery was similar regardless of whether or not the other parameter was free to vary in the model. This provides evidence that the estimation accuracy of each parameter was consistent no matter what other parameters were in the model, suggesting that the two parameters did not trade off against one another to a substantial degree during model fitting.

Added text in Results, page 10:

Supplementary Section S9 also reports the results of a parameter-recovery analysis, which indicated that all of the parameters in each model could be estimated with adequate accuracy by our model-fitting procedure.

Added text in Methods, page 30:

The parameter recovery analysis was performed by simulating 100 "participants" for each of the four primary models under consideration, with different parameter settings for each simulated "participant". For each model, we then quantified parameter recoverability as the Spearman correlation between actual and estimated parameter values for each free parameter.

Added text in Supplementary Material, Section S9:

We also conducted a parameter recovery analysis. Here, we simulated a behavioral dataset of 100 synthetic participants for each of the models presented in Table 1. To ensure that synthetic data were similar to the observed data, parameter values for synthetic participants were drawn from the estimated group-level posterior distributions from the models as fit to actual data. We then used the estimation procedure described in the main text to estimate all free parameters for the synthetic data generated from each model.

Parameter recovery results are presented in Table S7, and graphically in Figure S10 for the best-fitting Model 4. There are two noteworthy features of these results: first, parameters were recovered at acceptable (> 0.5 accuracy) levels for all parameters, albeit with less accuracy for φ and α_V than for the softmax inverse temperature parameter β. Second, for each parameter, estimation accuracy was similar across all models in which the parameter was fit (columns in Table S7), suggesting that the accuracy of estimation of each parameter was not substantially influenced by the simultaneous estimation of the other parameters in the model.

New Table S7 in Supplementary Materials:

Table S7: Summary of parameter recovery results for each of the models in Table 1.

Model	β	ϕ	α_V
1	.92	-	-
2	.86	.61	-
3	.93	-	.65
4	.94	.59	.64

Parameter recovery quantified as the Spearman rank-order correlation between generated and estimated parameter values.

New Figure S10 in Supplementary Materials:

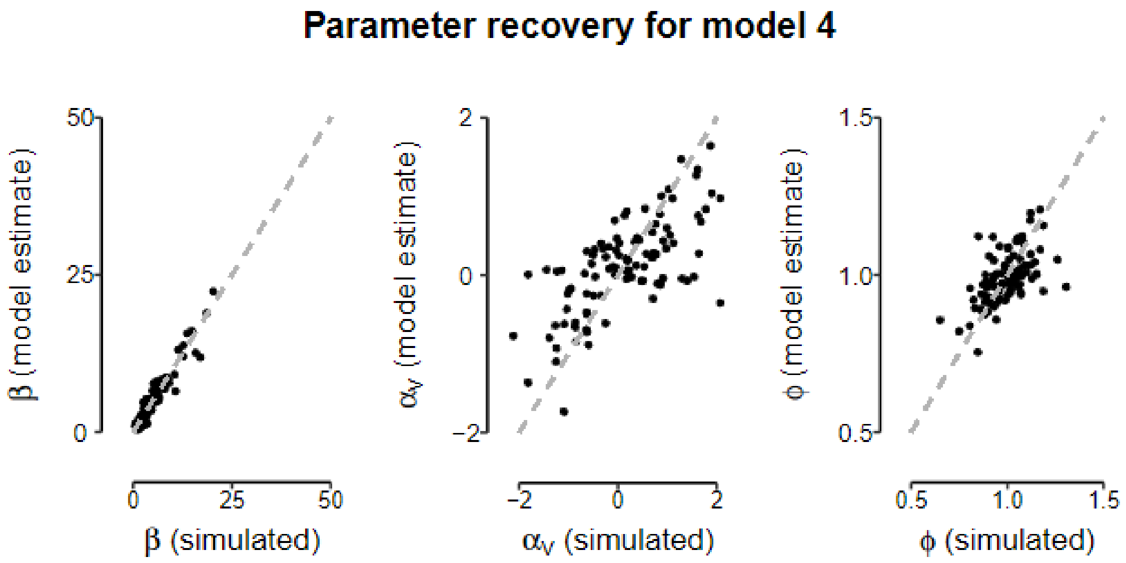

Figure S11: Parameter recovery results for each free parameter in Model 4. Under perfect parameter recovery, all points would fall on the grey x=y line.

R2.8 Continuing with interpreting the bias towards compound stimuli; does this preference suggest aversion to uncertainty was at play in your paradigm? If so, might this limit the generalizability of your findings to other situations in which affect-induced attentional biases emerge. Subjects ostensibly knew simple stimuli in key comparisons were 50/50, whereas all other stimuli had less outcome entropy (even if their average integration would yield the same level of outcome entropy). From my understanding, in the model, the effect of positive and negative emotion induction is equivalent but in opposing direction (which you tested thoroughly by comparing to other ways in which the inductions may have asymmetrical effects).

Might this not necessarily be the case if you de-confound preference for compound stimulus and uncertainty aversion? It could, for example, reveal that the effects of a negative affect induction is stronger than a positive affect induction, given that the preference towards the simple stimulus is now less uncertain (where that preference I believe indicates attention to negative features in the competing compound stimulus). I guess one way to implement this would be to focus on manipulating reward magnitude as opposed to probability.

We agree that aversion to uncertainty may have had an influence on participants' choice behaviour in the compound-generalisation task. However, investigating this possibility is made complicated by the fact that there are several different kinds of uncertainty at play in our task: first, at the cue level, there is uncertainty as to the strength of association between each cue and reward, as noted by the reviewer. For compound stimuli, however, decisions are subject to a higher-order form of uncertainty relating to the estimated value of a compound stimulus: since participants receive no instruction in how to interpret compound stimuli, there is inherent uncertainty as to how they should estimate the combined value of a compound containing simple cues with different reward values. As a construct, therefore, 'aversion to uncertainty' might be interpreted as predicting either increased preference for 25/75 compounds (if 'aversion to uncertainty' means aversion to the entropy of the 50% cues) or aversion to compounds more generally (if 'aversion to uncertainty' means aversion to the ambiguity inherent in compound stimuli). Unfortunately, it is not possible to resolve these competing hypotheses using the behavioral data that we collected in this task, and further research is required to understand how aversion to uncertainty might affect the cognitive processes underlying compound generalisation.

We thank the reviewer for raising this interesting question, which we now suggest as a potential future direction in the Discussion.

New text in Discussion, page 21:

> *In addition, further research is required to investigate the role aversion to uncertainty might play in compound generalization. In our task, there were at least three different forms of uncertainty that could have affected choice: first, at the cue level, there was uncertainty as to the probability of reward for each cue (often called ambiguity), as well as the actual receipt of reward given the probabilistic nature of the task ("risk," which was greatest for the 50% cue). Additionally, for compound stimuli, preferences may have been sensitive to uncertainty regarding how to estimate the value of a compound, since participants received no instruction on how to interpret these stimuli, and no feedback in the compound generalization trials. Future work could*

consider expanding the set of cue-reward contingencies tested in both simple and compound cues to shed further light on this question.

R2.9 Was there any evidence that subjects forgot the values of stimuli over the course phase 2 of the experiment, and specifically, whether subjects selectively forgot stimulus values conditional on the affect induction? I suppose it's possible that an affect induction could alter how we retain information in memory, which then goes on to predict what we attend to. I think an assumption of the current model is that one's current affect influences attention at the time of value formation. Is there a way to rule out that individuals merely were less certain about the affect-induction-incongruent stimuli, and thus didn't take those stimuli into account when making subsequent decisions (reflect in choice preference and eye gaze)?

The reviewer raises a good point. In interpreting the results of our experiment, we assumed that participants perfectly remembered the values of the simple cues throughout the compound-generalization phase. In other words, we assumed both that participants initially learned the simple-cue values successfully and that these learned values were not forgotten during the compound-generalization phase. Evidence for the former is provided both by Figure 2A (simple cue test accuracy), which suggests that participants successfully learned individual cue values, and Figure 5B, which suggests that attention (as measured with eye-tracking) scaled linearly with learned value, as predicted by evidence accumulation accounts of value construction. The strongest evidence for the second (retention) assumption comes from Figure 2C (generalization accuracy), which shows that in trials in which one option was better than the other, participants correctly combined simple cues to form compound values, and moreover, they did so in a manner that was not differentially impacted by affect. In addition, we found no evidence for an effect of block number on generalization accuracy during this phase of the task, which further supports the conclusion that participants did not forget the values of stimuli during the compound-generalization phase. The lack of an effect of block number on generalization accuracy is now reported in the revised manuscript:

In choices between compound stimuli with different mean cue values, participants tended to choose the stimulus with the higher mean value (mean choice proportion for higher-mean-value stimuli (SD) = 0.71 (0.13); $p < .001$, Wilcoxon test against chance). This pattern did not differ significantly across affect groups (β = -0.09, p = .38, mixed-effects logistic regression) or across different blocks of the task (β = -0.003, p = .95, mixed-effects logistic regression).

Nevertheless, it is possible that experienced outcomes (i.e. individual memories of cue->reward pairings) are differentially sampled (recalled) during compound generalization probe trials, and that this sampling process may be biased by affect. This form of “attention to memory” is not incompatible with our main hypothesis. In fact, we view these processes as two sides of the same coin - affect-biased external attention to the sensory environment or affect-biased internal attention to memory, both in service of generalization. While with our data we cannot precisely distinguish a bias in internal vs. external attention, we have added the following sentences in the Discussion to highlight this interesting distinction (page 22):

> *Finally, our results point to the influence of mood-congruent attentional biases when forming generalized reward expectations. How might such an attentional mechanism be implemented? Attention and memory-based processes both play a role in generalization (Radulescu et al., 2021). These accounts are not incompatible: because generalization by definition requires switching between (internal) attention to memory and (external) attention to the sensory environment, one way mood-congruent attentional biases could arise is by directing attention to memories as a function of their valence (Poskanzer and Aly, 2023; Gresch et al., 2023). This suggests it might be beneficial to integrate affective biases into sampling-based models of value-based and attribute-based attention.*

R2.10 Your work sheds new light on a prior finding that those who worry tend to perseverate on punishment avoidance goals (Sharp, Russek et al. 2022). There, subjects reporting high anxiety tended to incorporate learning about punishment avoidance features on trials where subjects were instructed to ignore such features. This would suggest that potentially attentional biases to threat, engendered by an anxious emotion, may override instructions to modulate attention. I think it may be worth mentioning this connection, given the potential of your model to extend to RL paradigms requiring individuals not only to attend to multiple features, but to explicitly not attend to certain features given instructed or learned goals. This could bolster your discussion on the ecological validity of your model — in the real world, not only do we need to attend to multiple features, but also alter how we prioritize stimulus features conditional on our shifting goals. That all said, I realize the conflict introduced by my suggesting this work (even if I did not de-anonymize myself, it is implied reviewers may part of suggested literature to cite), and so, I would like to reassure the authors that engaging this point here or in text won’t influence my judgment of their work in the least.

We thank the reviewer for this recommendation, which points out an interesting connection with the reviewer's work on multi-goal reinforcement learning. The suggested citation has now been included in the paragraph of the discussion in which we contextualise results with respect to computational models of affect (page 22):

> *Affective modulation of attention might also provide a useful framework for conceptualising the role of anxiety in value-based decision making, which has been shown to drive a bias to learn about punishment-avoidance features when the goal is to avoid threat rather than accrue reward.*

R 2.11 Figure S9 there's a reference to a Table that reads, "Table ??"

This LaTeX rendering error has been corrected in the revised manuscript.

R2.12 Last, I was surprised but inspired by the inclusion of the "citation diversity statement" at the article's conclusion. It's heartening to know this problem is not only being taken into account when considering citations, but also that the practice is being explicitly broadcast as a teaching signal to us all so that we can actually try to improve the current situation. Besides reading a thoughtful paper that advances science I intrinsically enjoy, I'm fortunate to have been selected as a reviewer of this paper to be exposed to this practice. I look forward to implementing it in my future work.

Thank you very much for acknowledging the effort to include a citation diversity statement. This is inspired by work by Dani Bassett and many other authors who now include such statements in their work. We are happy to contribute to such practices continuing to be adopted broadly, so we can all work together toward a shared goal of equity and inclusion in academia.

Reviewer #3

In this interesting study, the authors examine how positive and negative moods bias the evaluation of compound stimuli, whose value depends on a combination of attributes. The aim is to test a mechanistic account for a well-known finding (and intuition) that mood biases evaluation of events.

To do so, the authors use a task wherein participants choose between cues whose associated reward probabilities were learned previously, whilst also undergoing eye tracking. The authors focus on choices between medium-reward cues (either singly or in pairs) and pairs of high- and low-reward cues (H-L pairs).

The key behavioural findings are i) preference for the H-L pair depends on induced-mood, being higher following positive mood induction and lower following negative mood induction ii) relative time spent looking at the high probability cue is associated with higher choice of the H-L pair, and iii) for a subset of choices, mood induction increases the relative time spent looking at the mood-congruent cue.

The authors fit a computational model wherein compound stimuli are valued as a weighted average of their constituent cue values. Through model comparison they show that models in which the weighting parameter transiently changes following mood induction outperform alternatives without such modulation by mood induction. The authors interpret the weighting parameter as reflecting allocation of attention, and support this interpretation with reference to the eye-gaze findings. They suggest that this offers a mechanism by which mood modulates attention paid to mood-congruent features of events.

I found the study to be well thought-out and the manuscript well written. I appreciated the clear figures, which linked up nicely with the main text. The model fitting methods and statistical analyses appeared appropriate to test the hypothesis and were overall clearly described. Overall I thought the paper was impressive in its execution.

My only significant concerns relate to the interpretation of the results:

R3.1 The authors interpret their computational model in terms of relative allocation of attention. However they also acknowledge that a weighted contribution of cues could be interpreted in terms of other cognitive process. I think it could be emphasised more that it is difficult to disentangle effects of 'attention' from integration of value. For instance, it seems likely that gaze and value integration are co-dependent. (The authors do allude to this in the Discussion, with reference to sampling-based models but it could perhaps be foregrounded more).

This is an excellent point also raised by R2 (comment #9). For convenience, we re-iterate here the answer to R2's point, which specifically suggested forgetting of simple cue values as a potential alternative explanation for our findings:

In interpreting the results of our experiment, we assumed that participants perfectly remembered the values of the simple cues throughout the compound-generalization phase. In other words, we assumed both that participants learned the simple-cue values successfully initially and that these learned values were not forgotten during the compound-generalization phase. Evidence for the former is provided both by Figure 2A (simple cue test accuracy), which suggests that participants successfully learned individual cue values, and Figure 5B, which suggests that attention (as measured with eye-tracking) scaled linearly with learned value, as predicted by evidence accumulation accounts of value construction. For the latter assumption (retention of learned simple-cue values during the compound-generalization phase), the strongest evidence comes from Figure 2C (generalization accuracy), which shows that, in trials in which one option was better than the other, participants correctly combined simple cues to form compound values, in a manner that was not differentially impacted by affect. Nevertheless, it could be the case that experienced outcomes (i.e. individual memories of cue->reward pairings) are differentially sampled during probe trials, when both options have the same value and that this sampling process (which memories are recalled during compound generalization) may be biased by affect. This form of "attention to memory" is not incompatible with our main hypothesis. In fact, we view these processes as two sides of the same coin - external attention to the sensory environment or internal attention to memory, both in service of generalization. While with our data we cannot precisely distinguish a bias in internal vs. external attention, we have added the following sentences in the Discussion to highlight this interesting distinction (page 22):

> *Finally, our results point to the influence of mood-congruent attentional biases when forming generalized reward expectations. How might such an attentional mechanism be implemented? Attention and memory-based processes both play a role in generalization (Radulescu et al., 2021). These accounts are not incompatible: because generalization by definition requires switching between (internal) attention to memory and (external) attention to the sensory environment, one way mood-congruent attentional biases could arise is by directing attention to memories as a function of their valence (Poskanzer and Aly, 2023; Gresch et al., 2023). This suggests it might be beneficial to integrate affective biases into sampling-based models of value-based and attribute-based attention.*

We would like to further acknowledge that while at the time our data was written up for publication, a multi-attribute version of sequential sampling models was not readily available, recent advances in that domain potentially make it possible to now answer some of these follow-up questions using hierarchical drift diffusion modeling (Fisher, 2021; Chuan-Peng et al., 2022; Yang & Krajbich, 2023). While this is a direction we are very interested in pursuing, we opted to publish the initial hypothesis-driven results separately, and leave further exploratory analyses on the same dataset to future re-analysis.

R3.2 The authors state that their eye gaze findings suggest mood-dependent deployment of attention to the high vs low probability cues in compound stimuli. However they compute gaze-time relative to all other cues on the screen, rather than relative to the other cue in the high-low compound stimulus. The former metric (relative to all other cues) would increase with time spent looking at the high-low compound stimulus as a whole. In other words, if a subject were to spend more time looking at the high-low compound, apportioned equally between high and low cues, by the authors' metric, the proportion of time spent looking at the high cue would increase.

Thus, the authors cannot safely conclude that mood affects the allocation of attention to high vs low components of the compound stimuli themselves. This is important because relative attention assigned to H and L cues within the compound underpins the analogy to the computational model. As presented currently, it seems the findings could be explained if attention paid to the high-low compound - as a whole – varied following mood induction. This would be consistent with any model wherein mood increased the value of the high-low compound.

We thank the reviewer for bringing up these important points. To the reviewer's first question about how the normalization scheme (relative to all cues, or only relative to the other cue in the L/H compound) affects the overall pattern of results, we repeated the full analysis with normalization done within the L/H compound. In short, we found that the result in Figure 5C holds (attention to the H cue normalized within the L/H compound predicts choice), while the result in Figure 6 does not (the impact of affect on attention is not specific to the H cue normalized within the L/H compound, but rather arises from a more complex interaction of attention to H cue relative to all cues on the screen). These new results are detailed in response to the comment 3.3 below.

We also agree that the metric analysed (proportion of looking time to the mood-congruent cue within the compound as a proportion of all cues on the screen) could be affected by overall distribution of attention to the L/H compound. To rule out this explanation,

we ran an additional analysis testing whether proportion of looking time to the mood-*incongruent* cue (i.e., the high-value cue for the sad mood induction, the low-value cue for the happy mood induction) within the compound was affected by the mood induction in the same way. If, as the reviewer suggests, the reported effects were due to increased attention to the L/H stimulus more broadly, then we would expect to observe an increase in attention to the mood-incongruent cue within the compound as well as to the mood-congruent cue. We found that this was not the case: there was no significant effect of the affect induction (pre- versus post-) on attention to mood-incongruent cues ($\chi^2(1) = 0.04$, $p = .85$, likelihood-ratio test), and no interaction between the affect induction and probe trial type ($\chi^2(1) = 0.08$, $p = .78$, likelihood-ratio test). We therefore conclude that the observed effects of the mood induction on visual attention were likely to be specific to the mood-congruent cues, rather than being affected by attention to the L/H compound more broadly.

To better foreground the normalization scheme we used and to address the reviewer's next comment, we added the following clarifying language when introducing the eye-tracking analysis (page 15):

> *To provide mechanistic insight into how affect modulates feature-based attention and its effect on choice, we next turned to analyses of looking time in compound-generalization probe trials (that is, trials in which participants chose between the L/H compound and either a M or M/M stimulus). We specifically asked (1) whether looking time to the high-value cue (\textit{H}) relative to all other cues was predictive of choice, and (2) whether looking time to the high-value cue changed as a result of affect inductions.*

And we substantially expanded our discussion of the discrepancy between the modeling and eye-tracking analyses to discuss why the way that the cues were normalized might affect the observed pattern of results (page 20):

> *We also found that the expected pattern of affect-congruent attention in probe trials depended on whether the looking time measure of attention was computed relative to all cues on the screen, or only captured relative attention between the low and high-value cues within a compound. In our task, this discrepancy suggests a more complex interaction between affect and attention than a simple value-congruence account. For instance, it could be that negative affect increases attention to uncertain cues, thereby indirectly shifting attention away from the high-value cues (Anderson et al. 2019). Future work might seek to address this alternative explanation by using cues for which values are pre-established (and elicited through self-report or other methods) and not acquired through conditioning.*

R3.3 Also, with respect to the above, the results are described in a potentially misleading way. For instance on p15 the authors write (my italics):

"We next asked whether the general tendency to look at the low- or high-value cue within an L-H compound drives changes in choice. We used a mixed-effects analysis to test whether, in all probe trials, time looking at the high-value cue within an L-H compound stimulus predicted choice of that stimulus, controlling for main effects of affect condition. Indeed, we found that the more time people spent looking at the high-value cue relative to time looking at all other cues in the trial, the more likely they were to choose the L-H stimulus over an M or M-M stimulus of the same expected value…"

Here, "within an L-H compound" suggests time spent looking at the H cue relative to the L cue (i.e., H/(H+L)), but this isn't the metric that is tested. The authors should at least include the result of an analysis based on gaze time expressed as H/(H+L), even if this is non-significant (e.g. in a supporting figure).

We thank the reviewer for pointing out this potentially misleading phrasing. What we had meant was to qualify which high-value cue (the one within the L-H compound) but we can see how this can be misunderstood as qualifying the looking time. We have now edited this sentence to instead say:

> *Then we asked whether the general tendency to look at the high-value cue within an L/H compound predicts changes in choice. We used a mixed-effects analysis to test whether, in all probe trials, time looking at the high-value cue relative to all other cues predicted choice of the L/H compound. (now P. 16)*

To the reviewer's main point relating to the analysis in Figure 5C, we have also re-run the analysis with normalization done within the L/H stimulus, as requested. This analysis shows that the relative looking time within the L/H compound does indeed predict the tendency to choose the L/H compound ($\chi2(1) = 7.54$, $p = .006$), albeit this result is somewhat noisier than for looking time as calculated as a proportion of all time gazing at cues (compare to figure 5C), likely because it relies on fewer measurements on each trial:

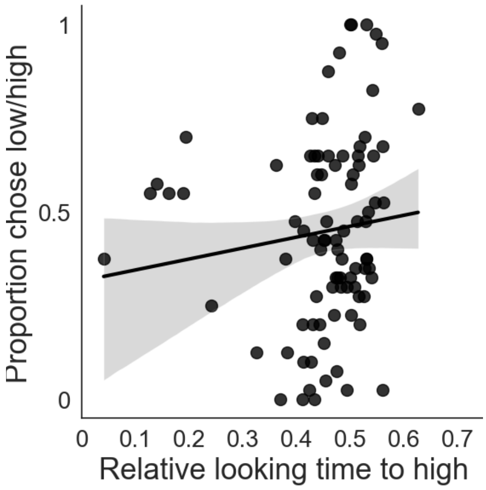

We have now included this additional result in the Supplementary Material, Section S11, and also updated the main manuscript text to more accurately reflect these findings:

> _Then we asked whether the general tendency to look at the high-value cue within an L/H compound drives changes in choice. We used a mixed-effects analysis to test whether, in all probe trials, time looking at the high-value cue relative to all other cues predicted choice of the L/H cue, controlling for main effects of affect condition. Indeed, we found that the more time people spent looking at the high-value cue, the more likely they were to choose the L/H stimulus over an M or M/M stimulus of the same expected value (Figure 5C, $\chi 2(1) = 18.6$, $p < .0001$, likelihood-ratio test for mixed-effects regression). This finding also held for relative looking time to the high-value cue as a proportion of total time looking at the L/H stimulus ($\chi 2(1) = 7.54$, $p < .01$, Supplementary Figure S12), suggesting that the effect on choice is specifically due to an increase in relative attention to the high-value cue within the compound._

We have also re-run the mixed-effects analysis pertaining to Figure 6 with normalization done within the L/H stimulus, and clarified in the main text that the results are not significant when running the analysis this way (page 18):

> _We note that this pattern of results was only evident when calculating looking time as a percentage of gaze to all cues present on the screen; when we instead calculated looking time to affect-congruent cues as a proportion of time looking at the L/H stimulus, we did not find a significant effect of the affect induction on looking time (see Supplementary Material, section S11 and Discussion)._

The previous analysis (Figure 5C) directly implicates a relative attention to the H cue over the L cue in driving choice regardless of affective state. However, the mixed-effects analysis suggests that how affect impacts overall attention to each cue is driven not only by an increase in attention to the H cue, but by a more complex pattern of shifts in attention

between all cues on the screen. We have now added a short paragraph in the discussion addressing this discrepancy between computational modeling and eye-tracking results (page 20):

> *We also found that the expected pattern of affect-congruent attention in probe trials depended on whether the looking time measure of attention was computed relative to all cues on the screen, or only captured relative attention between the low and high-value cues within a compound. In our task, this discrepancy suggests a more complex interaction between affect and attention than a simple value-congruence account. For instance, it could be that negative affect increases attention to uncertain cues, thereby indirectly shifting attention away from the high-value cues (E. C. Anderson, Carleton, Diefenbach, & Han, 2019). Future work might seek to address this alternative explanation by using cues for which values are pre-established (and elicited through self-report or other methods) and not acquired through conditioning.*

R3.4 The authors link eye gaze findings with choice proportions (% choose L-H). However, they don't test whether eye gaze is related to the key model parameter, alpha_v. This missing link would close the loop of the argument nicely. As things stand, the connection with the model is by analogy only. This perhaps makes the model fitting exercise appear a little redundant.

We thank the reviewer for this great suggestion! We have run this additional analysis and found that α_V significantly predicts relative looking time to the high-value cue:

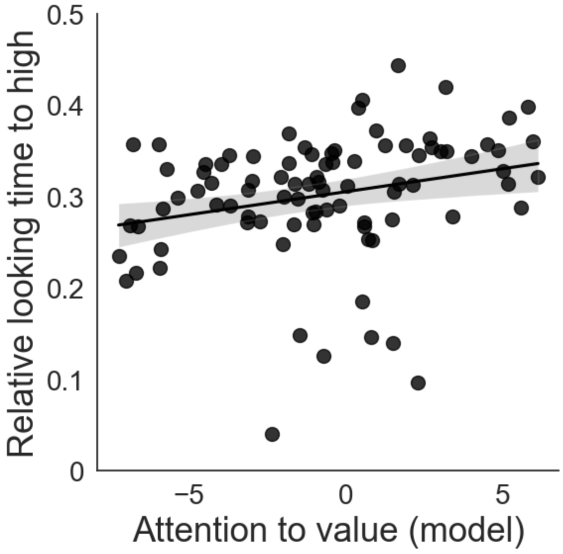

We have added this result as a figure in the main manuscript (Figure 5D) and added the following text to describe and interpret the finding (page 17):

To establish the correspondence between our two measures of attention to value, we ran a mixed-effects analysis with the model-based attention parameter as a predictor of relative looking time to the high-value cue. We found that the model-based attention to value (individually estimated from choice during all compound generalization trials, without regard to looking time) was a significant predictor of people's tendency to look at the high-value cue during probe trials (Figure 5D, $\chi^2(1) = 4.69$, $p < .05$). This result validates our choice of the model-based attention parameter as a metric of value-based attention. The result was not significant for relative looking time to the high-value cue within the L/H stimulus ($\chi^2(1) = 0.71$, $p = .41$, Supplementary Figure 12B), a point we return to in the discussion.

Minor Comments

R3.5 Figure 1 –very nice, but the cues appear quite small – not sure if there's a way to make them a bit larger or more contrasting, for display purposes?

We agree that cues in Figure 1 are rather small. The reason for this is that our goal for this figure was to accurately depict the visual proportions of stimuli on the screen as viewed by participants. We felt this was particularly important to convey since eye-tracking was an important component of our project. To address the reviewer's comment, therefore, we have revised Figure 1 to increase the contrast (as suggested by the reviewer) by increasing the brightness of the background grey rectangles in the task schematic. We feel that this change reflects an appropriate trade-off between the legibility of the manuscript and the accuracy of its depiction of the visual stimuli in the task.

R3.6 "positive, neutral or negative, between participants" On first reading I missed the fact the direction of mood induction was a between-subjects manipulation. The between subject aspect is mentioned only in brackets here and could perhaps be emphasised more.

We agree, and have added an additional sentence describing the overall between-participants experimental design at the beginning of the Results section.

New text in Results, page 3:

The experiment used a between-participants design for affect inductions, with participants pseudo-randomly allocated to receive either positive, neutral, or negative affect inductions during the task.

R3.7 Several results are given as beta coefficient estimates with p-values – it's not clear without referring to the Methods what kind of regression each refers to. The authors might for instance briefly state the type of analysis, at the start of each section e.g. mixed effects, between subjects etc. Also, later in the Results section the authors state F stats and partial eta squared, while here no test statistics are given.

We thank the reviewer for identifying this oversight. In the revised manuscript, we report the statistical analysis that has generated each test statistic and *p*-values alongside each statistic. The previously unlabelled statistics identified by the reviewer were all produced by mixed-effects regression analyses (either logistic or linear, depending on whether the dependent variable for the analysis was binary or continuous). Due to the many places where we have now added these descriptions, we do not replicate them here, however, several examples were already evident in the modified text included in response to other concerns.

R3.8 P7 - The wording when describing weighted averages is unclear: "Based on previous studies of compound generalization, and given no explicit instruction on how to estimate the values of these new stimuli, we assumed that participants would estimate the value of a compound stimulus as a weighted average of the values of the cues within the compound (Rescorla, 1976; McLaren & Mackintosh, 2002, one alternative to which is a non-linear conjunction rule; c.f. Saavedra, 1975; Holland, 1991). A qualitative prediction of such a linear rule is that the value of a compound stimulus should be proportional to the mean of the values of its constituent cues.." It seems misleading that "such a linear rule" follows the previous clause, which mentions a "non-linear rule".

We agree that this sentence was awkwardly phrased in context, and have edited this paragraph to address this issue in the revised manuscript (see also response to comment R3.10 below).

Updated text, page 7:

Based on previous studies of compound generalization, and given no explicit instruction on how to estimate the values of these new stimuli, we assumed that participants would estimate the value of a compound stimulus as a weighted average

of the values of the cues within the compound (Rescorla, 1976; McLaren & Mackintosh, 2002~~, one alternative to which is a non-linear conjunction rule; c.f. Saavedra, 1975; Holland, 1991~~). A qualitative prediction of ~~such a linear~~ <u>a weighted-average</u> rule is that the value of a compound stimulus should be proportional to the mean of the values of its constituent cues (e.g. an L/H [25/75] stimulus should be treated as more valuable than an L/M [25/50] stimulus, but less valuable than an M/H [50/75] stimulus).

R3.9 "A qualitative prediction of such a linear rule is that the value of a compound stimulus should be proportional to the mean of the values of its constituent cues". Doesn't this prediction encounter a limit as the weighting parameter for any one of the cues (A_j) approaches 1?

We agree that this is an important limiting case for the weighted-average model in general (though one that we did not encounter in our data; see response to Reviewer 2, comment R2.6). We have added an additional footnote in the revised manuscript detailing this caveat.

Added footnote, page 7:

[1] <u>This prediction encounters a limit as the weighting on any single cue approaches 100%, in which case the value of the compound would be solely determined by the value of the single attended cue.</u>

R3.10 "These findings support the explanation that participants estimated compound cue values using a linear weighted average". I'm not sure I follow this. Since the task only examines compounds involving up to two cues, how can non-linear and linear weightings be distinguished? Is not the case that either model boils down to a single parameter (A_1, as expressed below in my comment with reference to the model definition)?

Our primary intention in the quoted sentence was to emphasise that participants appeared to compute the value of compound cues as a weighted average of the values of simple cues. The non-linear combination rules that we are able to rule out on the basis of these findings are primarily non-linear conjunctive rules, such as assigning a high value to a compound if it contains *either* a high-value cue *or* a low-value cue (i.e., an XOR rule). In the revised manuscript, we have added additional material clarifying this point.
Updated text in Results, page 7:

Based on previous studies of compound generalization, and given no explicit instruction on how to estimate the values of these new stimuli, we assumed that participants would estimate the value of a compound stimulus as a weighted average of the values of the cues within the compound (Rescorla, 1976; McLaren & Mackintosh, 2002~~, one alternative to which is a non-linear conjunction rule; c.f. Saavedra, 1975; Holland, 1991~~). A qualitative prediction of ~~such a linear~~ a weighted-average rule is that the value of a compound stimulus should be proportional to the mean of the values of its constituent cues (e.g. an L/H [25/75] stimulus should be treated as more valuable than an L/M [25/50] stimulus, but less valuable than an M/H [50/75] stimulus).

Updated text in Discussion, page 20:

Although weighted cue combination is a common assumption across models of generalization (e.g., Rescorla & Wagner, 1972; Pearce, 1987), we note that participants were not instructed regarding how to interpret compound stimuli, and never experienced outcomes associated with these stimuli. As such, they could conceivably have chosen other strategies, such as evaluating the compound using a non-linear XOR rule (e.g., a compound with either cue A or cue B has high value), as is observed in the biconditional discrimination paradigm (Saavedra, 1975). Within the set of possible weighted cue combination rules, we found that participants' behavior during compound generalization was more consistent with cue averaging than with cue summation. This is notable because cue averaging is predicted by configural models of compound generalization (e.g., Pearce, 1987), which assume that compounds are treated as unique configurations that accrue value according to their similarity to the values of other stimuli (the simple cues, in this case). By contrast, elemental models of compound generalization assume that expectations for compound stimuli are computed by summing the values of independent features (Rescorla & Wagner, 1972; Melchers et al., 2008; Duncan, Doll, Daw, & Shohamy, 2018; Radulescu, Niv, & Ballard, 2019). Here, elemental models would predict that participants would never prefer simple stimuli over compound stimuli (since the summed value of compound cues was always equal to or greater than the value of simple cues in our task). More broadly, whether behavior is consistent with elemental or configural generalization has been shown to depend on experimental context (for review see Soto et al., 2014; Kehoe, Horne, Horne, & Macrae, 1994). In our case, the binary reward distribution that participants were exposed to in the training phase ~~cover story, which suggested that probabilities of outcome could change across~~

~~planets, but not the amount of outcome available,~~ *likely favored configural (linear combination) generalization over elemental summation.*

R3.11 "There was no evidence for an association between any individual-difference measure and performance in either probe trial type, and no interaction between any individual difference measure and the strength of the effect of the affect induction on choice behavior (all p > .10)"_Perhaps there could be a reference to supporting material or Methods here for a list of individual difference measures tested.

We have made the suggested revision.

Updated text, page 8:

There was no evidence for an association between any individual-difference measure and performance in either probe trial type, and no interaction between any individual difference measure and the strength of the effect of the affect induction on choice behavior (all p > .10; see Methods for a list of individual-difference measures).

R3.12 Model definition. The model is presented in general form – however, for compounds of two cues it reduces to:_V(compound) = A_1*v_1 + A_2*v_2 Where A_2=1-A_1.

We agree, and have added this detail in a new footnote in the revised manuscript.

New footnote in revised manuscript:

[2] *Equation 1 presents the model in its general form. For compounds of two cues like those in the present study, this equation can also be expressed as V(compound) = $\phi(\alpha_1 v_1 + (1-\alpha_1)v_2)$.*

R3.13 Figure 3: What is the scale for the horizontal axis for the alpha_v parameter here? Equation 2 suggests that alpha_v can be unbounded, yet the authors place a vertical dotted line at alpha_v=0, suggesting that the plot shows alpha_v for the high valued cue relative to the low value cue. The description of the results on p11 also suggests this relative scaling of alpha_v.

The scale for α_V is its natural (unbounded) scale, not a difference between high- and low-value cues. The vertical dotted line at $\alpha_V = 0$ in Figure 3 reflects the 'null' value of α_V; that is, the value of α_V at which there is no modulation of attention to cues as a function of

their value (because of the functional form of Equation 2, positive values of α_V produce greater attention to high-value cues and negative values produce greater attention to low-value cues). In the revised manuscript, we have added additional text to the legend of Figure 3 to clarify this point.

Updated figure legend for Figure 3:

> *Estimated posterior distributions for group-mean parameters. Top histograms: group-level means for the parameters α_V (A), ϕ (B), and β (C). Bottom histograms: group level means for the effect of affect on α_V (D), the effect of affect on ϕ (E), and the block-wise decay in the strength of these effects, $\boldsymbol{\lambda}$ (F). Horizontal error bars denote the 90% credible interval for each parameter, and vertical dotted lines denote the reference value for each parameter (i.e., the parameter value at which there is no influence of the parameter on behavior: 0 for additive parameters such as α_V, 1 for parameters that were multiplicative or exponents such as ϕ and $\boldsymbol{\lambda}$). Asterisks denote parameters for which there is credible evidence (i.e., estimated probability in excess of 95%) that the group mean is different from the reference value.*

R3.14 Figure 4: This shows decay over time as predicted by the model. It would be nice to see how the model fits to the observed data. For instance could the authors promote Figures S8a and S8b to the main text, and show model fits to the observed pattern of change across blocks?

We agree that it would be of interest to visualise the capacity of the model to capture the time-dependent effects captured in Figures S8a and S8b. We have therefore added an additional figure to the Supplementary Material of the revised manuscript in which we illustrate that the best-fitting model is indeed able to capture this pattern of time-dependent effects (Figure S9). We have not promoted these figures to the main text from the Supplementary Material due to the length of the manuscript, which already includes six figures and three tables. We would of course be willing to revisit this decision if the reviewer and editor feel it is important.

Updated text in Results, page 13:

> *Finally, the posterior distribution of the mean of the decay parameter $\boldsymbol{\lambda}$ was significantly smaller than one (posterior median = 0.45; Figure 3F), suggesting a considerable block-by-block reduction in the strength of affect's influence on behavior.*

<u>As a result, the best-fitting model was able to capture the time-dependent effects of the affect induction on behavior (see Figure S9)</u>.

R3.15 Figure 5: Here the plotted markers for the medium reward cue are rather faint.

Thank you for this suggestion, we regenerated that figure to have clearer markers for the group means.

R3.16 Figure 5c: The authors show fitted lines for each mood induction group, that suggest an interaction. However, they don't refer to analysis of a [group x looking-time] interaction in the Main Text. Incidentally I'm not convinced about the interaction, which seems to rest on one data point, but would be worth stating if this was tested or not.

We thank the reviewer for catching this omission, and for correctly estimating the effect. We now mention that the interaction is not significant in the text (page 16):

(*<u>Figure 5C, $\chi^2(1) = 18.6$, $p < .0001$, likelihood-ratio test for mixed-effects regression; interaction was not significant: $\chi^2(1) = 3.7$, $p = .16$)</u>*

And we updated the figure to illustrate the main effect:

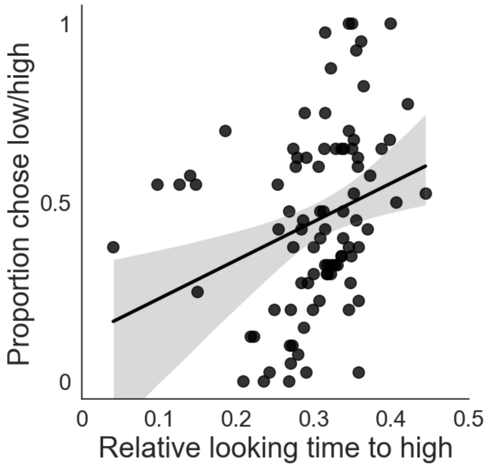

R3.17 In the paragraph on p15 referring to Figure 5c, the authors state a chi-squared statistic with only one-degree of freedom – is this correct? And why is a chi-squared test used here?

This is correct: a chi-square test is used here because the underlying statistical test by which significance is tested for these regressors in a mixed-effects regression is a *likelihood-ratio test* (in which the test statistic is distributed according to a chi-square distribution under the null hypothesis). This detail has now been added to the revised manuscript as part of our

efforts to provide additional detail on the statistical tests in the manuscript (prompted by the reviewer in comment R3.7). As with our response to R3.7, due to the many places where we have now added these descriptions of likelihood-ratio tests, we do not quote them here.

R3.18 The authors asked "whether the general tendency to look at the low- or high-value cue within an L-H compound drives changes in choice". However, from the analysis we can't really establish a causative relationship between gaze and choice, since gaze time may also be a function of the value integration process.

Thank you for this comment. We have updated the wording to reflect the correlational nature of the analysis (page 16):

> *Then we asked whether the general tendency to look at the high-value cue within an L/H compound ~~drives~~ predicts changes in choice.*

R3.19 Figures 5c vs. 6: The authors could more clearly draw out, in their description of these two figures and associated analyses, whether comparisons are between- or within-subjects. For instance in Figure 5c is there a single data point per subject?

Thank you, we added the following clarification to the caption for Figure 5c (page 16):

> *Data points are color-coded by condition (neutral, positive and negative affect). Each point corresponds to one participant in each of the three groups.*

R3.20 The authors refer here to 'simple probe trials'. However earlier in the Results section a terminology such as 'simple vs simple' and 'simple vs compound' was used. On reading the Methods it appears these are different ways of referring different kinds of probe trial – a consistent terminology would make things clearer for the reader.

We thank the reviewer for raising this point, and apologise for the unnecessary confusion. To clarify:

- "Simple versus simple" refers to a trial in which the participant was given the choice between two simple stimuli
- "Simple versus compound" refers to a trial in which the participant was given the choice between one simple stimulus and one compound stimulus

- “Simple probe trials” refers to a kind of simple-versus-compound trial in which the participant chooses between a 50%-valued simple stimulus and a 25%/75% compound stimulus
- “Compound probe trials” refers to a kind of compound-versus-compound trial in which the participant chooses between a 50%/50% compound and a 25%/75% compound.

In the revised manuscript we explicitly define the terms in the list above that were not already defined (see first paragraph on page 8, final paragraph on page 15, and Figure 1C).

R3.21 “Taken together, these results suggest a potential computational and perceptual-level mechanism for the well-documented finding that positive affect is associated with optimistic future expectations”. The results do outline a putative mechanism– namely selective attention to affect congruent stimulus features. With a particularly critical reading, it could be argued that the model is simply an alternative description of “the well-documented finding that positive affect is associated with optimistic future expectations”, especially given concerns over the interpretation of the eye-gaze data. Overall however, I think the study is interesting, and that the formalism lends itself to further investigation of how mood might bias attention to different stimulus attributes in a valence-dependent way. The conclusions might be strengthened by examining a link between relative gaze time and the attention weights derived from the model.

We are very thankful to the reviewer for suggesting the analysis in 3.4, which we ran and added to the main manuscript as a datapoint supporting the claim that our model-estimated attentional parameter is a significant predictor of relative looking time to the high-value cue.

Given that our eye-tracking results did depend on the normalization scheme, we have significantly expanded our discussion to account for potential alternative explanations of our findings, and hope the reviewer will be satisfied with the balance in interpretation (page 19):

> *Our study operationalized attention in two different ways. On the one hand, we operationalized attention as a parameter (α_V) within the computational model of behaviour that controlled the degree to which cues within a stimulus contributed to the overall value of that stimulus. This is equivalent to the common `attention weight' formalization of attention in models of choice (see, e.g., Kruschke, 2001). On the other hand, in our eye-tracking analysis, we operationalized attention as the proportion of total looking time spent looking at each cue during the choice phase (Leong et al. 2017; Krajbich et al. 2010; Krajbich et al. 2011, Krajbich et al. 2012;*

Callaway et al. 2019). Our results showed some discrepancies between the model-based measure of attention and the eye-tracking measure of attention.

Most notably, we found an unexpected pattern of results when trials were subdivided into simple probe trials (involving choices between one single-cue stimulus and one compound-cue stimulus) versus compound probe trials (involving choices between two compound stimuli). Consistent with the behavioral and computational modelling results, relative time looking at affect-congruent cues (i.e., looking time for high-value cues after a positive affect induction and for low-value cues after a negative affect induction) increased as a result of the affect induction. This effect was seen in compound probe trials, but surprisingly was not present in simple probe trials. Given that our eye-tracking results normalised attention across all the cues present on the screen, one potential explanation for this unexpected distinction is that compound stimuli pose a more stringent requirement for overt attention (i.e., shift of eye gaze) due to visual crowding (Whitney et al. 2011), whereas simple stimuli are more readily processed using covert attention only (see, e.g., Poder et al. 2006; Vater et al. 2017). As a result, our eye-tracking measure may have provided a better estimate of attention in trials where both stimuli were compounds.

We also found that the expected pattern of affect-congruent attention in probe trials depended on whether the looking time measure of attention was computed relative to all cues on the screen, or only captured relative attention between the low and high-value cues within a compound. In our task, this discrepancy suggests a more complex interaction between affect and attention than a simple value-congruence account. For instance, it could be that negative affect increases attention to uncertain cues, thereby indirectly shifting attention away from the high-value cues (Anderson et al. 2019). Future work might seek to address this alternative explanation by using cues for which values are pre-established (e.g. via self-report) and not acquired through conditioning.
